# Presence of Extensive *Wolbachia* Symbiont Insertions Discovered in the Genome of Its Host *Glossina morsitans morsitans*

**DOI:** 10.1371/journal.pntd.0002728

**Published:** 2014-04-24

**Authors:** Corey Brelsfoard, George Tsiamis, Marco Falchetto, Ludvik M. Gomulski, Erich Telleria, Uzma Alam, Vangelis Doudoumis, Francesca Scolari, Joshua B. Benoit, Martin Swain, Peter Takac, Anna R. Malacrida, Kostas Bourtzis, Serap Aksoy

**Affiliations:** 1 Department of Epidemiology of Microbial Diseases, Yale School of Public Health, New Haven, Connecticut, United States of America; 2 Department of Natural Sciences, St. Catharine College, St. Catharine, Kentucky, United States of America; 3 Department of Environmental and Natural Resources Management, University of Patras, Agrinio, Greece; 4 Dipartimento di Biologia e Biotecnologie, Università di Pavia, Pavia, Italia; 5 Department of Biological Sciences, McMicken College of Arts and Sciences, University of Cincinnati, Cincinnati, Ohio, United States of America; 6 Institute of Biological, Environmental and Rural Sciences, Aberystwyth University, Penglais, Aberystwyth, Ceredigion, United Kingdom; 7 Institute of Zoology, Section of Molecular and Applied Zoology, Slovak Academy of Science, Bratislava, Slovakia; 8 Biomedical Sciences Research Center Al. Fleming, Vari, Greece; 9 Insect Pest Control Laboratory, Joint FAO/IAEA Division of Nuclear Techniques in Food and Agriculture, Vienna, Austria; National Institute of Allergy and Infectious Diseases, United States of America

## Abstract

Tsetse flies (*Glossina* spp.) are the cyclical vectors of *Trypanosoma* spp., which are unicellular parasites responsible for multiple diseases, including nagana in livestock and sleeping sickness in humans in Africa. *Glossina* species, including *Glossina morsitans morsitans* (*Gmm*), for which the Whole Genome Sequence (WGS) is now available, have established symbiotic associations with three endosymbionts: *Wigglesworthia glossinidia*, *Sodalis glossinidius* and *Wolbachia pipientis* (*Wolbachia*). The presence of *Wolbachia* in both natural and laboratory populations of *Glossina* species, including the presence of horizontal gene transfer (HGT) events in a laboratory colony of *Gmm*, has already been shown. We herein report on the draft genome sequence of the cytoplasmic *Wolbachia* endosymbiont (cytWol) associated with *Gmm*. By *in silico* and molecular and cytogenetic analysis, we discovered and validated the presence of multiple insertions of *Wolbachia* (chrWol) in the host *Gmm* genome. We identified at least two large insertions of chrWol, 527,507 and 484,123 bp in size, from *Gmm* WGS data. Southern hybridizations confirmed the presence of *Wolbachia* insertions in *Gmm* genome, and FISH revealed multiple insertions located on the two sex chromosomes (X and Y), as well as on the supernumerary B-chromosomes. We compare the chrWol insertions to the cytWol draft genome in an attempt to clarify the evolutionary history of the HGT events. We discuss our findings in light of the evolution of *Wolbachia* infections in the tsetse fly and their potential impacts on the control of tsetse populations and trypanosomiasis.

## Author Summary

African trypanosomes are transmitted to man and animals by tsetse fly, a blood sucking insect. Tsetse flies include all *Glossina* species with the genome of *Glossina morsitans morsitans* (*Gmm*) being sequenced under the International Glossina Genome Initiative. The endosymbionts *Wigglesworthia glossinidia*, *Sodalis glossinidius* and *Wolbachia pipientis* (*Wolbachia*) have been found to establish symbiotic associations with *Gmm*. *Wolbachia* is known to be present in natural and laboratory populations of *Glossina* species. In this study we report the genome sequence of the *Wolbachia* strain that is associated with *Gmm*. With the aid of *in silico* and molecular and cytogenetic analyses, multiple insertions of the *Wolbachia* genome were revealed and confirmed in *Gmm* chromosome. Comparison of the cytoplasmic *Wolbachia* draft genome and the chromosomal insertions enabled us to infer the evolutionary history of the *Wolbachia* horizontal transfer events. These findings are discussed in relation to their impact on the development of *Wolbachia*-based strategies for the control of tsetse flies and trypanosomiasis.

## Introduction

The genus *Wolbachia* encompasses intracellular maternally inherited Gram-negative bacteria estimated to infect over 40% of insect species, in addition to filarial nematodes, crustaceans, and arachnids [1], [2]. *Wolbachia* interactions with its host can have diverse outcomes that range from mutualistic to pathogenic or reproductive parasitism [3]. In arthropods, *Wolbachia* alterations to host reproduction include parthenogenesis induction, male killing, feminization of genetic males, and cytoplasmic incompatibility (CI) [1], [4]. In its simplest form, CI occurs when a *Wolbachia* infected male mates with an uninfected female, causing developmental arrest of the embryo. In contrast, *Wolbachia* infected females can mate with either an uninfected male or a male infected with the same *Wolbachia* strain, and produce viable *Wolbachia* infected offspring. It has been suggested that the reproductive advantage afforded by the *Wolbachia* induced CI mechanism may permit the rapid spread of desirable host phenotypes into natural populations as a novel disease control approach [4]–[7].

A number of *Wolbachia* whole genome sequence (WGS) data are available to date and at least ten more genomes are currently being sequenced from a diverse set of hosts [8]–[15]. The majority of the *Wolbachia* strains have genomes that range from 1.08 to 1.7Mb in size [12]. Although most Rickettsiales have small genomes, *Wolbachia* sets a different pace by carrying an extremely high number of mobile and repetitive elements [4], [16], [17]. In addition, a number of Ecdysozoan genomes have been reported to contain chromosomal insertions originating from *Wolbachia*, including the mosquito *Aedes aegypti* [18], [19], the longhorn beetle *Monochamus alternatus* [20], filarial nematodes of the genera *Onchocerca*, *Brugia*, and *Dirofilaria* [21], [22], parasitoid wasps of the genus *Nasonia* [22], the fruit fly *Drosophila ananassae* [22], the pea aphid *Acythosiphon pisum* [23], and the bean beetle *Callosobruchus chinensis* [23], [24]. Horizontal gene transfer (HGT) events in prokaryotes are rather common, and represent a way for bacteria to acquire novel features that enable them to adapt to different environments and to reorganize their genome [25]–[27]. In unicellular eukaryotes, gene transfer events are also relatively common [28]. Since many unicellular eukaryotes are phagotrophic on bacteria and other micro-organisms, they are constantly exposed to prokaryotic DNA, which may predispose them to incorporate foreign genetic material into their genomes [29]. By contrast, in multi-cellular organisms HGTs are rare [30]. It is likely that the localization of *Wolbachia* within the host germ-line cells [31] may have enabled the transfer of its genetic material to the host chromosomes.

Tsetse flies are the exclusive vectors of Human African Trypanosomes (HAT), also known as sleeping sickness, and of the livestock disease Nagana in sub-Saharan Africa. These diseases are caused by different members of the kinetoplastid protozoan parasites, *Trypanosoma spp*. The World Health Organization (WHO) has estimated that 60 million people in Africa live in tsetse infested areas, and are at risk of contracting sleeping sickness [32]. Disease control in the mammalian host is complicated due to the lack of vaccines, cheap and effective therapeutic treatments, and simple accurate diagnostic tools [33], [34].

Tsetse flies also harbor multiple symbiotic microbes, which display different levels of integration with their host. The obligate mutualist genus *Wigglesworthia* provides dietary supplements to support host fecundity and is also necessary during larval development for the adult immune maturation processes [35]–[38]. The facultative symbiont genus *Sodalis* is present in some individuals in natural populations and may play a role in tsetse's trypanosome transmission ability (vector competence) [38], [39]. The ability to cultivate *Sodalis in vitro* and transform and repopulate tsetse with modified *Sodalis* has led to a potential paratransgenic control strategy to modify tsetse's vector competence by expressing trypanocidal molecules in rec*Sodalis* [40]–[44]. Natural populations of many tsetse species also harbor a third symbiont, which belongs to the genus *Wolbachia*. Recent surveys indicate that *Wolbachia* infection prevalence in natural populations of different tsetse species can vary considerably, with some populations having near 100% infection prevalence [41], [45]. We recently demonstrated that *Wolbachia* infections in *Glossina morsitans morsitans* (*Gmm*) induce CI in the laboratory and confer a reproductive advantage to infected females [41]. Further modeling of CI demonstrated the potential use of *Wolbachia* to drive a desirable host phenotype into a natural tsetse population [41], [46]. Thus, it is suggested that tsetse carrying modified *Sodalis* expressing antiparasitic molecules in their midgut can be used to replace their wild parasite-susceptible counterparts through *Wolbachia*-mediated CI. One population control method that has been successful for tsetse, and currently being implemented in Africa, is the sterile insect technique (SIT), where males rendered sterile through irradiation are released to mate with wild females and suppress their fecundity [41], [47]. A promising alternative/complementary approach to SIT could be the use of the incompatible insect technique (IIT), which relies on *Wolbachia-*induced sterility in the released males instead of irradiation [48], [49].

In this paper, which is being submitted as a satellite to the manuscript describing the WGS of the tsetse species *Gmm*, we report on the draft genome sequence of its associated cytoplasmic *Wolbachia* endosymbiont (cyt*Wol*). Moreover, we mined the WGS of *Gmm* and report on the presence of multiple extensive chromosomal insertions of *Wolbachia* (chr*Wol*) in the host genome. These results confirmed our previous PCR-amplification based data suggesting the presence of HGT event(s) between *Wolbachia* and *Gmm* [45]. The HGT events were validated by Southern blot and Fluorescent *in situ* Hybridization (FISH) analyses on *Gmm* chromosomes. We compared the chr*Wol* insertions discovered in the assembled *Gmm* genome to cyt*Wol* to understand the evolution of HGT events, and discuss our findings in light of the evolution of *Wolbachia* infections in tsetse. Finally, we analyzed the presence of *Wolbachia* HGT events in several *Gmm* natural populations, and discuss the potential to harness *Wolbachia* effects for the control of tsetse-transmitted diseases.

## Materials and Methods

### Cytoplasmic *Wolbachia* source DNA and sequencing

For the genome sequencing of the naturally infected *Wolbachia* strain of *G. m. morsitans* (*w*Gmm), approximately 250 ovaries were dissected from adult females from the *Gmm* colony maintained in the Yale University insectary. DNA was prepared using Qiagen DNeasy kit (Qiagen, Inc., Valencia, CA). The complete genome sequence was determined using whole-genome shotgun pyrosequencing using the Roche 454 GS sequencer FLX Titanium system (454 Life Sciences, Branford, CT, USA).

In order to improve the *w*Gmm draft genome, Illumina read libraries from the tsetse genome assembly were used. These were obtained from: (a) a pool of five tsetse flies. and (b) the first larval progeny of tetracycline-treated female. Two sets of Illumina reads were used: a PCR-free small fragment (∼300 bp) library and Hi-Seq mate-pair libraries with an insert of approximately 1.6 kb.

### Cytoplasmic *Wolbachia* assembly and annotation

The tsetse ovary DNA used for *w*Gmm sequencing contained a mixture of host genetic material, as well as cytoplasmic (cyt) and chromosomal (chr) *Wolbachia* DNA. A customized informatics pipeline was developed to computationally distinguish between sequence reads. An initial assembly was performed using MIRA [50]. First, all host sequences were removed by mapping the 454 reads to the *Wolbachia* reference genomes (*w*Mel, *w*Ri, *w*Pip and *w*Bm). The filtered sequence reads contained chromosomal and cytoplasmic reads. The chromosomal reads were further removed using MIRA by mapping the filtered sequences to the chromosomal *Wolbachia* contigs (99% cut-off). The same procedure was followed for the Illumina data. The resulting 454 and Illumina reads were *de novo* assembled using MIRA. This initial assembly was subsequently improved using approaches described in the PAGIT protocol [51]. In brief, the contigs were aligned to the *w*Mel genome using ABACAS [52], creating one large scaffold that consisted of the contigs successfully mapped to the *w*Mel genome and a set of contigs that did not map. An attempt was made to close the gaps in the large scaffold using IMAGE [53] with the PCR-free small fragment library. After gap closing, the large scaffold was reduced once more to a set of contigs by breaking it around any of the unclosed gaps. This is because there are usually many genome rearrangements between different *Wolbachia* strains, and we would therefore expect a number of rearrangements to exist between the *w*Mel and *w*Gmm genomes. Breaking the scaffold makes allowance for these gaps. Finally, scaffolding was then performed on this reduced set of contigs using SCARPA [54] with the Hi-Seq mate-pair libraries. The statistics for the assembly at each stage of the process are given in Table S1.

The genome was annotated with XBASE and RAST [55], [56], followed by manual curation. Putative protein-encoding genes were identified using GLIMMER [57] and tRNA by tRNAscan-SE [58]. Predicted proteins were examined to detect frame-shifts or premature stop codons to identify pseudogenes using ARTEMIS [59]. Those for which the frame-shift or premature stops were of high quality by examining re-mapped reads in these regions were annotated as “authentic” mutations. This Whole Genome Shotgun project has been deposited at DDBJ/EMBL/GenBank under the accession AWUH00000000. The version described in this paper is version AWUH01000000.

### Chromosomal *Wolbachia* assembly and annotation

The Sanger and 454 reads used in the tsetse genome assembly were obtained from flies treated with tetracycline as described previously [41]; therefore, these reads did not contain cyt*Wol* sequences. As mentioned above, *Wolbachia* specific sequences were filtered out from WGS reads of each sequencing technology with MIRA [50] using the complete genomes of *w*Mel (AE017196), *w*Ri (CP001391), and *w*Bm (AE017321) as reference sequences. We obtained 5,306 (Sanger), and 10,978 (454) *Wolbachia*-specific sequences respectively. All the filtered putative *Wolbachia*-specific sequences were further examined using blast and a custom made *Wolbachia* database.

Chr*Wol*-specific sequences were assembled with MIRA and AMOS [50], [60] using as a reference sequence the *w*Gmm draft genome. The statistics for the two chr*Wol* assemblies are as follows: N50 2970, mean contig length 1261.97, longest contig 15053, total length 527,504 bp for insertion A, while for insertion B N50 2791, mean contig length 1092.82, and total length 484,123. Genes were identified with Glimmer [61], followed by a round of manual curation using Blastn [62] and MegaBlast [62] against the non-redundant and custom made *Wolbachia* databases. The predicted CDSs were translated and used to search the NCBI non-redundant database, KEGG, and COG databases. The tRNAScan-SE tool [58] was used to identify tRNA genes.

### Phylogenetic analyses

Phylogenetic analyses were performed using Maximum-Likelihood (ML) and Neighbor-Joining (NJ) estimation for a concatenated set of six phage genes from *w*Gmm, *w*Ri, *w*Mel and *w*Pip. The genes used for the phylogeny included *HK97 family phage major capsid protein* (*w*Gmm_0882, WD_0458, WRi_002750, WP0102), *phage integrase family site-specific recombinase* (*w*Gmm_0004, WD_1148, WRi_009900, WP0980), *phage SPO1 DNA polymerase-related protein* (*w*Gmm_0674, WD_0164, WRi_000900, WP0922), *prophage LambdaW5 baseplate assembly protein W* (*w*Gmm_0971, WD_0640, WRi_005480, WP0303), *prophage LambdaW1, baseplate assembly protein J* (*w*Gmm_0970, WD_0639, WRi_010130, WP0302) and a *prophage LambdaW1, site-specific recombinase resolvase protein* (*w*Gmm_0960, WD_0634, WRi_005400, WP0342).

In addition, a concatenated set of ten genes (*DNA-directed RNA polymerase, DNA polymerase III (alpha subunit), DNA gyrase B, translation elongation factor G, aspartyl-tRNA synthetase, CTP synthase, glutamyl-tRNA(Gln) amidotransferase B, GTP-binding protein, cell division protein FtsZ, fructose-bisphosphate aldolase*) from the identified *Gmm* chromosomal insertions, *w*Gmm, *w*Ri, *w*Mel, *w*Pip, and *w*Bm were used.

All sequences were aligned using MUSCLE [63] and ClustalW [64] as implemented in Geneious 5.4 [65], and adjusted manually. ML and NJ trees were constructed using MEGA 5.0 [66] with gamma distributed rates with 1000 bootstrap replications and the method of Tamura-Nei as genetic distance model [67].

### Southern blot hybridization analyses

To determine the number of chromosomal insertions, genomic DNA from tetracycline-treated *Gmm* females and normal *Gmm* individuals were restricted with *Hind*III endonuclease, electrophoresed on 1% agarose gel in 1× TBE buffer, and transferred to a positively charged nylon membrane according to Southern protocol [68]. The membrane was hybridized at 55°C with 350 ng of a 569 bp probe corresponding to part of the *wsp* gene labeled with the Gene Images Alkphos Direct labeling system (GE Healthcare, Little Chalfont, UK) using the random primer method following manufacturer protocols. Signal detection was performed using CDP-star followed by exposure to autoradiographic film (X-OMAT AR, Kodak). The absence of cyt*Wol* from the tetracycline-treated *Gmm* DNA was confirmed by a PCR assay, which resulted in only a single *16S rRNA* amplification product originating from the chromosomal insertions [45].

### FISH chromosomal preparations and hybridization

Mitotic chromosome spreads were obtained from freshly deposited larvae from the Slovakia Academy of Sciences Institute of Zoology tsetse laboratory *Gmm* strain. Briefly, larval nerve ganglia were incubated on a slide in 100 µl 1% sodium citrate for 10 min at room temperature, and sodium citrate was replaced with methanol-acetic acid (3∶1 solution) for 4 min. The tissue was disrupted by pipetting in 100 µl 60% acetic acid for fixation and dropped onto clean slides heated on a hot plate at 70°C until acetic acid evaporation. After dehydration in 80% ethanol, slides were stored at −20°C for at least 2 weeks.

For *in situ* hybridization experiments, multiple probes specific for *Wolbachia* 16S *rRNA*, *fbpA* and *wsp* genes were amplified from the Slovakian strain DNA [45], [69]. To generate the labeled probes, 1 µg of DNA resuspended in 16 µl ddH_2_O was denatured by boiling for 10 min. 4 µl of labeling mix (Biotin High Prime kit; Roche, Basel, Switzerland) were added and the reaction was incubated overnight at 37°C. After the reaction was stopped, ddH_2_O (5 µl), 20×SSC buffer (25 µl) and formamide (50 µl) were added and 25 µl of denatured probe was placed on each pre-treated slide. The hybridization was performed at 37°C overnight in a humid chamber and detection of hybridization signals was performed using the Vectastain ABC elite kit (Vector Laboratories, Burlingame, CA, USA) and Alexa Fluor 594 Tyramide (Invitrogen). Chromosomes were DAPI stained and the slides were mounted using the VECTASHIELD mounting medium (Vector Laboratories). Chromosomes were screened under an epifluorescence Zeiss Axioplan microscope and images were captured using an Olympus DP70 digital camera. For the localization of signals on mitotic chromosomes the karyotype description of Willhoeft [69], [70] was adopted.

### Analysis of HGT fragments in *Gmm* genome via PCR and sequencing

Natural samples of *Gmm* used to examine HGT fragments originated from four populations collected in Zambia, Zimbabwe and Tanzania (Table 1). DNA was isolated from adult flies stored in EtOH using the Qiagen DNeasy kit (Qiagen, Valencia, CA) following the manufacturers' instructions and stored at −20°C. The aposymbiotic (*Wolbachia*-free) *Gmm* line [41] was used as a control. For detection of *Wolbachia*, a PCR assay that amplified a 438 bp *16S rRNA* fragment was used with the specific primer set wspecF and wspecR [71]. For input DNA control, a 377 bp fragment of the mitochondrial *12S rRNA* gene was amplified with the primer set 12SCFR and 12SCRR [72]. The PCR amplification protocol was 10 min at 95°C, 35 cycles of 30 sec at 95°C, 30 sec at 54°C and 1 min at 72°C, and 10 min at 72°C.

**Table 1:** Identity of analyzed *G. m. morsitans* samples.

Code	Country (Area, Collection Date)	Number of analyzed individuals	*cyt*Wol present
12.3A	Zambia (MFWE, Eastern Zambia, 2007)	43	41
30.9D	Zimbabwe (Rukomeshi, 2006)	50	42
32.3D	Zimbabwe (Makuti, 2006)	32	28
34.7G	Tanzania (Ruma, 2005)	44	35

The identification of the *Wolbachia* strain infections was based on MLST (*gatB*, *coxA*, *hcpA*, *fbpA* and *ftsZ*) and *wsp*-based genotyping approaches [45], [69]. PCR reactions were performed using the following program: 5 min of denaturation at 95°C, followed by 35 cycles of 30 sec at 95°C, 30 sec at the appropriate temperature for each primer pair (52°C for *ftsZ*, 54°C for *gatB*, 55°C for *coxA*, 56°C for *hcpA*, 58°C for *fbpA* and *wsp*) and 1 min at 72°C. All reactions were followed by a final extension step of 10 min at 72°C. Both strands of the products were sequenced using the respective primers. In addition, PCR products of *16S rRNA*, *wsp* and MLST genes from the *Gmm* populations analyzed were cloned in pGEM-T Easy Vector System, and PCR products from several clones generated by the primers T7 and SP6 were sequenced in both directions using the BigDye Terminator v3.1 Cycle Sequencing Kit (PE Applied Biosystems) and were analysed using an ABI PRISM 310 Genetic Analyzer (PE Applied Biosystems). All *Wolbachia* gene sequences were manually edited with SeqManII by DNAStar and aligned using MUSCLE [63], as implemented in Geneious 5.4 [65], and adjusted manually.

### Recovery of *Wolbachia* reads from RNA-seq data sets

To determine if genes from the chromosomal insertions were potentially expressed in locations other than the gonotrophic tissues, we utilized mapping of Illumina datasets from other studies, that included transcriptome reads from somatic tissues [73]–[75]. Reads were mapped to the chromosomal insertions using CLC Genomics Workbench (CLC Bio, Cambridge, MA) allowing no mismatches per reads, a maximum of 10 hits per read and 80% of the gene must match at 95%. Predicted open reading frames (ORF) from the insertions were extracted and the following criteria were utilized to determine the possibility of expression: 1) at least 25 reads were recovered from the ORF and 2) those represented had coverage of over 85% of the ORF. This filtering approach excluded genes with a high number of mapped reads that were only present in small limited sections of the ORFs. These sections with high read numbers mapping but low coverage could be where sequence similarity between *Gmm*, *Wigglesworthia* or *Sodalis* is high enough to yield mapping to the chromosomal insertions.

## Results

### Cytoplasmic *w*Gmm genome features

**Figure 1:**
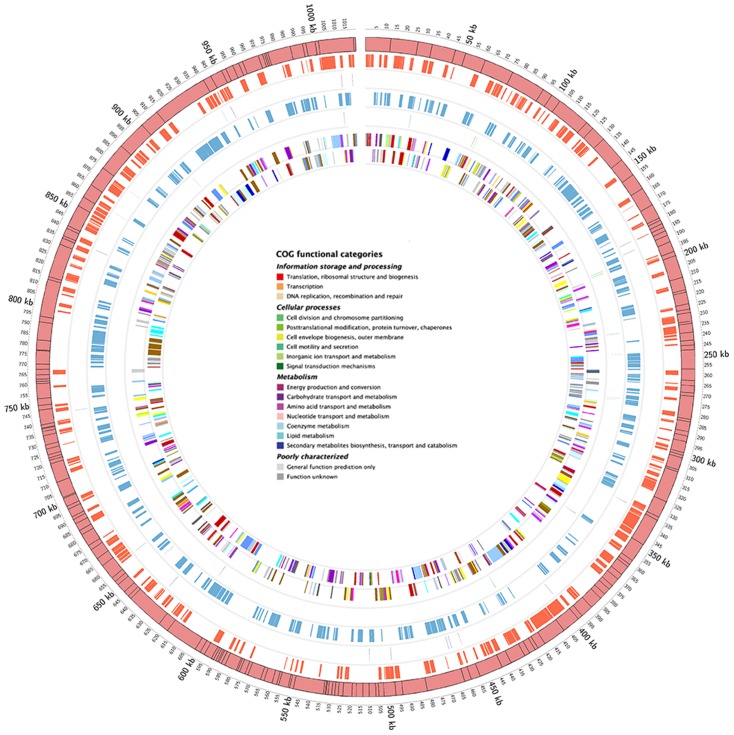
Circular map of the cytoplasmic *w*Gmm genome. The outermost circle represents the scale in Kbp. Contigs of the draft genome are presented as boxes and they have been randomly ordered into a single circle for presentation purposes. In the second and fourth circle CDS in the two strands are presented. In the third and fifth circle the position of the tRNAs are presented. In the sixth and seventh circle CDSs identified for the *w*Gmm genome are colored according to the Clusters of Orthologous Groups (COG) categories and represented as lines and boxes.

The draft genome of cytoplasmic *wGmm* contains 201 contigs of 1,019,687 bp, comprised of 800 putative functional coding sequences (CDS) and 16 pseudogenes (Figure 1 and Table 2). The GC content of *w*Gmm is 35.2%, in the range observed for the other sequenced *Wolbachia* genomes (Table 2). Although, the *w*Gmm genome is not complete, based on comparison of the identified contigs, it is most similar to the two *Wolbachia* strains associated with *Drosophila melanogaster* and *D. simulans*, *w*Mel and *w*Ri, respectively (Table S2). It is more distantly related to the genomes of the *Wolbachia* strains associated with *Culex pipiens* and *Brugia malayi*, *w*Pip and *w*Bm, respectively (Table S2). The majority of the regions and genes missing from the *w*Gmm genome relative to the *w*Mel and *w*Ri genomes encode phage, ankyrin and hypothetical proteins (Tables S3 and S4).

**Table 2:** *w*Gmm genome features and comparisons with other sequenced *Wolbachia* genomes.

Host	*Glossina morsitans*	*Drosophila melanogaster*	*Drosophila simulans*	*Brugia malayi*	*Culex pipiens*
*Wolbachia*	*w*Gmm	*w*Mel	*w*Ri	*w*Bm	*w*Pip
Genome size	1,019,687	1,267,782	1,445,873	1,080,084	1,482,355
G + C content (%)	35.2	35.2	35.2	34	34.2
% coding DNA	64.6	81	77.7	67.4	82
Predicted functional protein-coding genes	800	1,195	1,150	805	1,275
Pseudogenes	16	74	114	98	110
IS elements	10	32	67	33	62
IS – Mobile elements (% of genome)	1.2	8.9	22.1	5.4	7.1
Genes containing ankyrin repeats	10	23	35	5	60
Phage and prophage genes	14	38	13	?	118
Phage – prophage regions	Not Known	3 (2×WO-A and 1× WO-B)	4(1×WO-A, 2×WO-B, 1×WO-C)	0 prophage	5 (5× WO-B)
tRNA	34	34	34	34	34
23s rRNA	1	1	1	1	1
16s rRNA	1	1	1	1	1
5s rRNA	1	1	1	1	1

#### Repetitive and mobile DNA

One interesting feature of *Wolbachia* genomes is the presence of high numbers of genes encoding proteins containing ankyrin repeat domains (ANK), which are thought to play an important role in host-symbiont interactions, the establishment of symbiosis and the induction of reproductive phenotypes [76]. In comparison to the closely related *w*Mel and *w*Ri genomes, which contain 23 and 35 such genes respectively, the draft genome of *w*Gmm has only 10 genes encoding proteins with one or more ANK repeat domains exhibiting the highest sequence identity with *w*Mel, *w*Ri, and *w*Pip (Table 3).

**Table 3:** Ankyrin-domain containing proteins encoded by the *w*Gmm genome.

Locus		Annotation	Size (bp)	Contig number	Present (highest homology first)
*w*Gmm	0356	ankyrin repeat-containing prophage LambdaW1	1968	42	wPip, wMel, wRi
*w*Gmm	0372	ankyrin repeat-containing protein	1245	44	wMel, wRi
*w*Gmm	0536	ankyrin repeat containing protein	867	72	wMel, wRi
*w*Gmm	0561	ankyrin repeat-containing protein	255	76	wRi, wMel
*w*Gmm	0573	ankyrin repeat-containing protein	2082	78	wRi, wMel
*w*Gmm	0691	ankyrin repeat-containing protein	807	99	wMel, wRi
*w*Gmm	0870	ankyrin repeat-containing protein	1191	139	wMel, wRi, wPip
*w*Gmm	0967	ankyrin repeat containing protein	462	164	wPip, wMel, wRi
*w*Gmm	0968	ankyrin repeat-containing prophage LambdaW1	750	164	wPip, wRi, wMel
*w*Gmm	1071	ankyrin repeat-containing protein	1113	176	wRi, wMel

An additional feature of the *Wolbachia* genomes is the presence of a high number of repeat sequences, IS elements and prophages. However, the draft *w*Gmm genome contains a much reduced number of repeat elements, 1.2% compared to 8.9% in *w*Mel and 22.1% in *w*Ri, respectively (Table 2). This is could be due to assembly issues in the draft *w*Gmm assembly i.e. collapsed or unassembled repeats. The *w*Gmm contains only 10 IS elements made up of the following families: IS3, IS5, and ISwPi6 (Table 4). Only 14 phage related genes (partial or putatively protein encoding genes) were discovered in the *w*Gmm genome, a relatively small number when compared with *w*Mel, *w*Ri, and *w*Pip. Phylogenetic analysis based on six concatenated phage genes suggested that the *w*Gmm phage genes are more closely related to the *w*Mel and *w*Ri than the *w*Pip corresponding phage (Figure S1).

**Table 4:** IS-elements identified in the *w*Gmm genome.

Locus		Family	contig
*w*Gmm	0025	IS5	002
*w*Gmm	0026	IS5	002
*w*Gmm	0027	IS5	002
*w*Gmm	0028	IS5	002
*w*Gmm	0191	IS3	014
*w*Gmm	0310	IS5	031
*w*Gmm	0628	ISWPi6	082
*w*Gmm	1201	Transposase Tn5 related	0187
*w*Gmm	1200	Transposase Tn5 related	0187
*w*Gmm	0961	Putative uncharacterized	0163

#### General comparison with other *Wolbachia* genomes

Comparisons of *w*Gmm, *w*Mel, *w*Ri, and *w*Bm suggest that a high degree of rearrangement has occurred in the multiple genomes. There are many blocks of genes that share co-linearity with *w*Ri, *w*Mel and *w*Bm. While several of the genomes have undergone extensive rearrangements, the co-linear blocks are most likely maintained due to their important biological functions and co-transcription. An example that has already been discussed in the literature [10], [77] is the type IV secretion system (T4SS), for which the gene order function is also conserved in *w*Gmm (Figure S2).

### Chromosomal *Wolbachia* features

**Figure 2:**
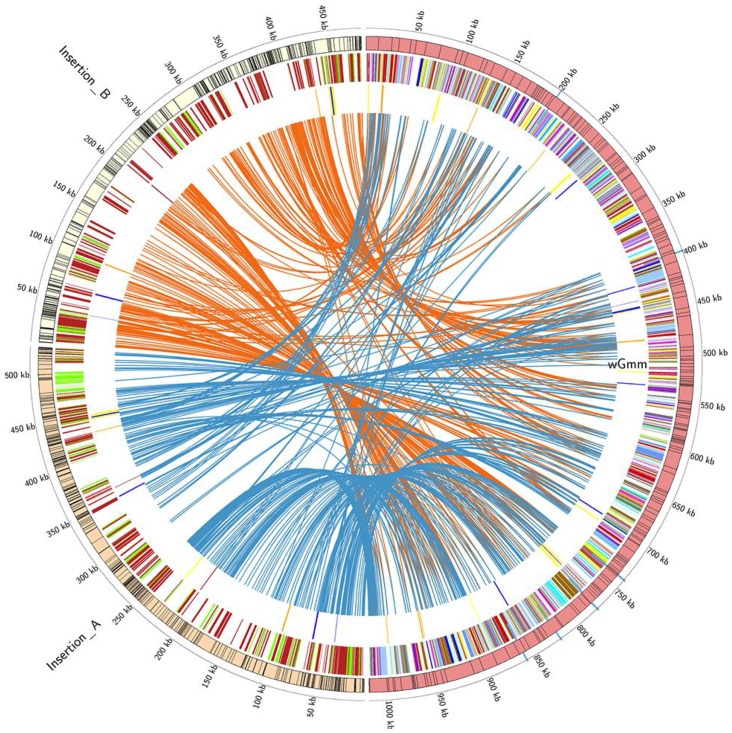
Circular map of the *w*Gmm genome (red) and the chromosomal insertions in *Gmm* genome (orange and light yellow). The outermost circle represents the scale in Kbp. Contigs comprising the two identified chromosomal insertions are presented as boxes while the position of the third insertion relatively to the *w*Gmm contigs is presented as blue boxes just below the outmost circle. In the second circle CDSs identified for the *w*Gmm genome are colored according to the Clusters of Orthologous Groups (COG) categories and represented as lines and boxes. Genes identified in the insertions (orange and light yellow) of *w*Gmm in the tsetse fly genome are represented as lines and boxes. Pseudogenes are presented in red while in green coding DNA. Circles three presents the position of ankyrins (blue), prophages (yellow) and transposons (orange), in *w*Gmm, and the chromosomal insertions. Finally, the *w*Gmm genome and the two insertions in the tse-tse fly genome are arranged around the circle, with bands connecting regions of homology. Blue ribbons are composed of synteny regions, identified using Mauve and Mummer 3.0, between *w*Gmm genome and the first set of identified insertions in the tsetse genome. Orange ribbons are composed of synteny regions, identified using Mauve and Mummer 3.0, between *w*Gmm genome and the second set of identified insertions in the tsetse genome.

Both PCR-based evidence from *Wolbachia* infected tsetse flies, and analysis of the *Gmm* annotated genome data indicated the presence of *Wolbachia* gene fragments inserted in the host genome. We mined the final assembly of the *Gmm* host genome and were able to identify 261 contigs that carried chr*Wol* DNA sequences. Based on nucleotide diversity, close examination of the 261 contigs indicated that these represented at least three different events, which we refer to as insertions A, B and C. Manual editing and implementation of the AMOS snps script enabled the separation of the contigs into different insertions, with insertions A and B being the largest in size. Figure 2 shows the mapping of these two insertions on the *w*Gmm reference genome. The observed pattern suggests that at least two large *Wolbachia* genome segments of 527,507 and 484,123 bps have been integrated into the *Gmm* chromosomes indicating that at least 51.7% and 47.5.% of the draft *Wolbachia* genome were transferred to the host nuclear genome. Sequence analysis of insertion A predicted 197 putative functional coding sequences, 148 pseudogenes, and 15 tRNAs. Remnants of 163 pseudogenes were discovered that are greater than 100 bp in size and that have either partially been integrated into the host genome, or only represent part of the pseudogene. For insertion B, sequencing analysis revealed the presence of 159 putative functional coding sequences, 148 pseudogenes and 13 tRNAs. In insertion B, 157 remnants of pseudogenes were also identified. Thus, on average more than 60% of the genes transferred to the tsetse nuclear genome have been pseudogenized. The average length of the putative functional coding sequences is slightly smaller than *w*Mel, *w*Ri and the cytoplasmic *w*Gmm at 690 bp for insertion A and 677 bp for insertion B (Table S4). The GC% content for insertion A and B is 35.1%. Comparison between the chromosomal insertions A and B and the *w*Gmm draft genome using Blastn and lastz indicated that: (a) the two insertions are very similar to each other (Figure S4) and (b) at least four genes, three hypothetical proteins and *hemK* are present in the chromosomal insertions but not in the cytoplasmic *Wolbachia* genome. The sequence identity between chromosomal and cytoplasmic genes and phylogenetic analysis based on ten concatenated genes clearly suggests that the chromosomal insertions A and B are closely related to the cytoplasmic *w*Gmm genome (Table 5 and Figure S3). In more detail, comparison of the sequence identity in eleven chromosomal genes indicates that the majority of them exhibit a high sequence identity with the *w*Gmm sequences (Table 5). The third *Wolbachia* HGT segment, insertion C, is only 2,089 bp in size and sequence analysis predicted the presence of only six pseudogenes.

**Table 5:** Sequence identity between chromosomal and cytoplasmic genes.

Insertion set A (coding genes)	*w*Gmm	*w*Ri	*w*Mel	*w*Pip
Type IV secretion protein virB10	98.3	87.1	95.9	78
Type IV secretion protein virB11	99.7	98.6	98.3	88
prophage LambdaW1	98.5	80.7	87.5	91.4
ankyrin repeat-containing gene	97.9	67	80.7	67.8
prophage LambdaW5 site-specific recombinase resolvase	99.5	84.9	87.5	92.5

Insertion set B (coding genes)	*w*Gmm	*w*Ri	*w*Mel	*w*Pip
Type IV secretion protein virB8	99.1	93.2	98.7	88.4
Type IV secretion protein virB10	99.1	83.5	98.5	77.1
Type IV secretion protein virB11	99.7	98.3	98	87.7
Type IV secretion protein virB6	99	98.7	95.8	90
ankyrin repeat-containing prophage LambdaW1	99.6	88.1	88.5	90.5
prophage LambdaW5	99.7	81.2	81.2	97.9

**Figure 3:**
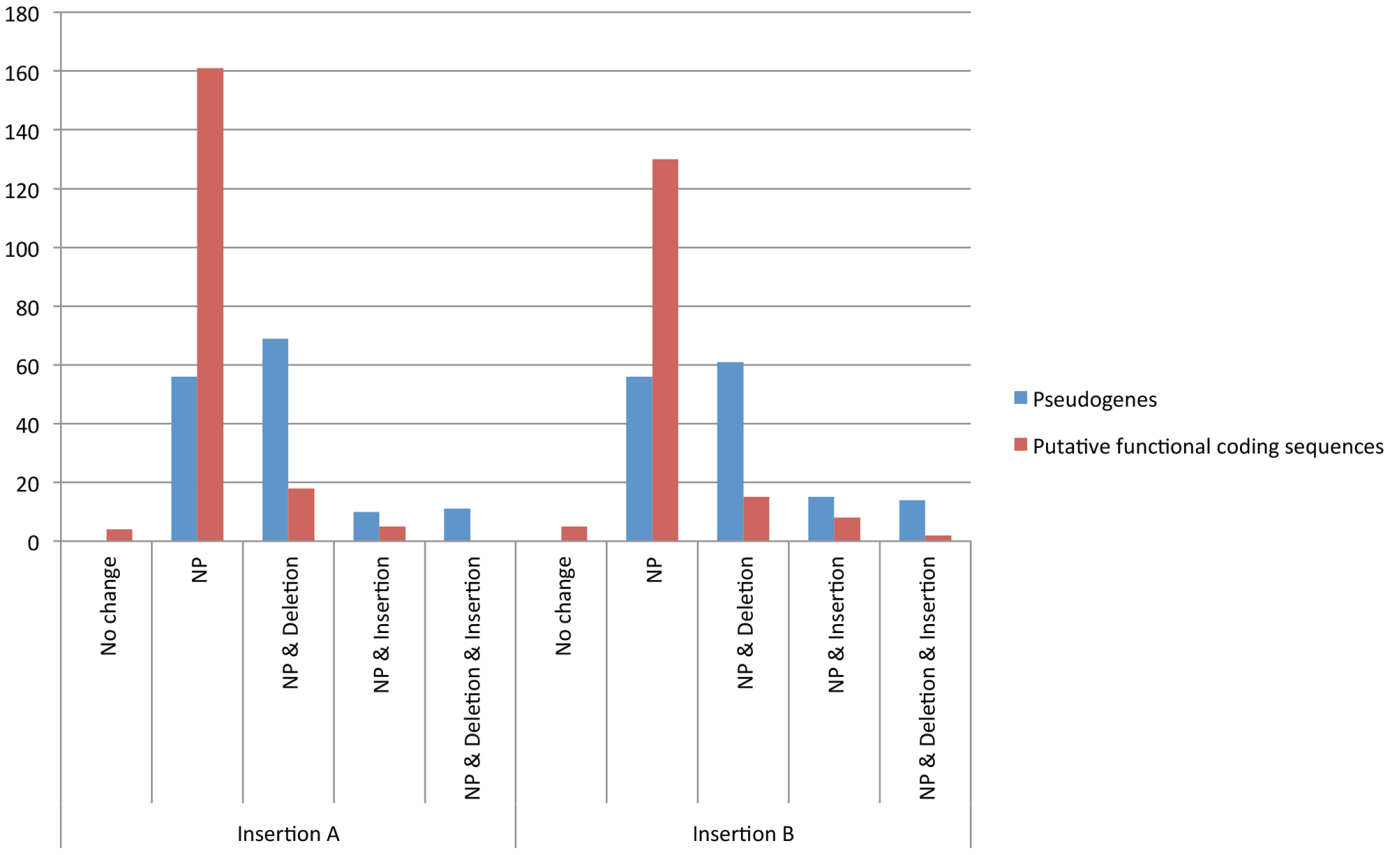
*w*Gmm chromosomal genome properties in terms of the number of identified pseudogenes (blue) and putative functional coding sequences (red).

A number of different types of mutations were identified in insertions A and B present in the host nuclear genome, and these shed light on the pseudogenization process. Our analysis suggests that more than 80% of the mutations that accumulated in the putative functional coding sequences represent single nucleotide polymorphisms (SNPs) (Figure 3). The majority of the genes that have been pseudogenized accumulated mutations that consist of nucleotide polymorphisms with deletions (NPD) and NPs. In both insertions, genes that have been pseudogenized contain mutations that combine NPs and deletions (NPDs) are more than those pseudogenized by NPs (Figure 3). In addition, we identified two additional types of mutations, NPs with insertions and NPs with deletions and insertions, associated with both chromosomal *Wolbachia* insertions but to a much lesser degree. A list of partial and full genes corresponding to the chr*Wol* insertions is available in Tables S6, S7 and S8.

### Expression of chromosomal sequences

Based on our results, there were very few ORFs that met our criteria for expression from chromosomal insertions. In general, there were multiple ORFs that had high number of mapped reads (>100), but in nearly all cases the coverage of the mapping was below 30% indicating that these may represent reads from another symbiont or tsetse transcripts. Results were similar for the three transcriptomes analyzed from heads, salivary glands and the bacteriome. However, three putative ORFs satisfied our criteria: *serB*, *ccmB* and a degenerate transposase located at both insertions (102636-102894 for insertion A and 97255-97523 for insertion B). These analyses suggest that most of the genes present in the chromosomal insertions are likely not expressed, but the few specific genes we identified may have low levels of expression. Further studies will be necessary to validate their expression.

### Southern blot analysis

**Figure 4:**
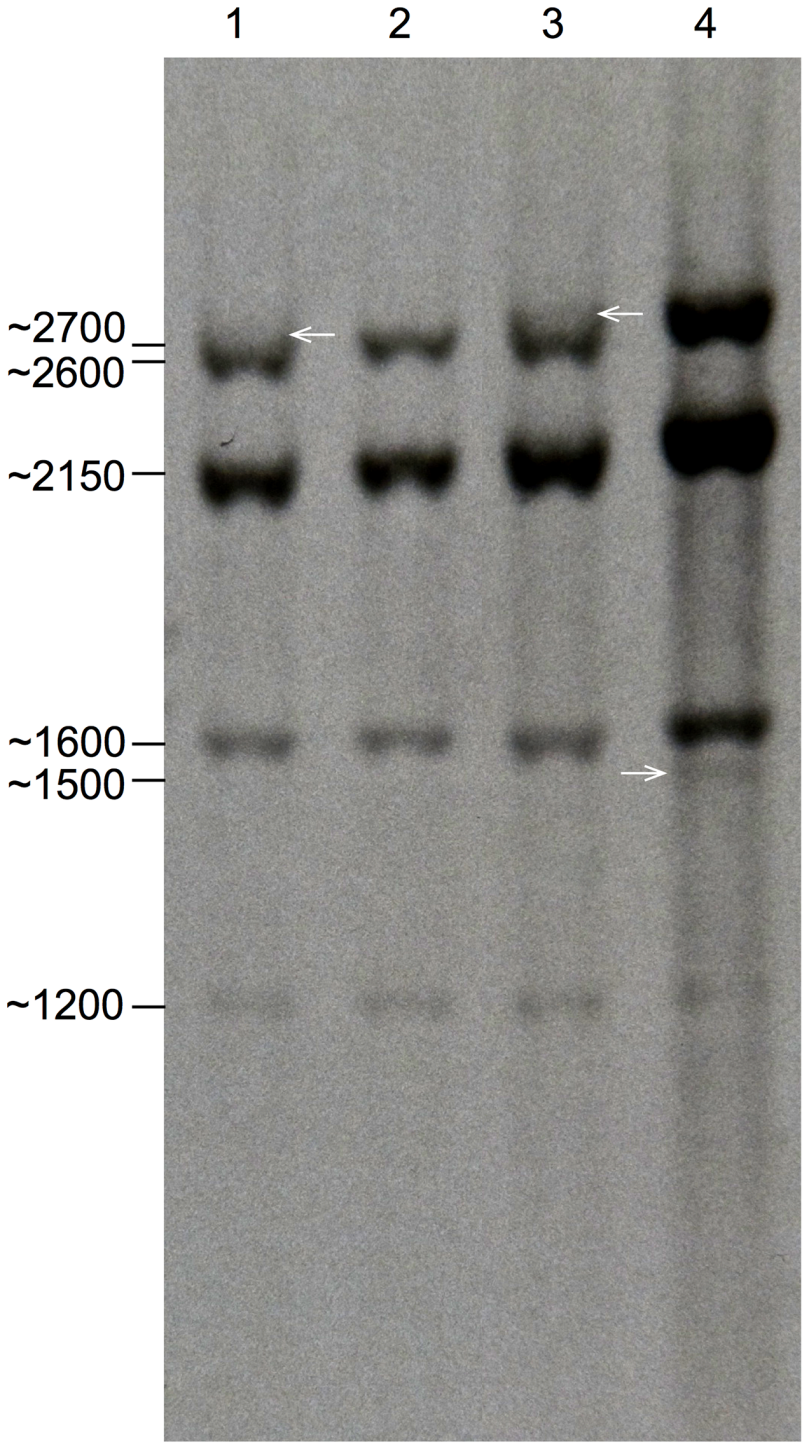
Southern blot analysis showing *wsp* hybridizing fragments. DNA cleaved with *Hind*III restriction enzyme from normal females (lanes 1 and 3), tetracycline-treated females (lane 2) and normal males (lane 4) are shown. The sizes of the hybridizing bands are shown. The ∼2700 bp band (indicated by arrows) may represent the cytoplasmic *wsp* containing fragment (lanes 1 and 3) which is absent in the tetracycline treated females (lane 2). The ∼1500 bp band present only in the male (lane 4) is indicated by an arrow.

Hybridization of the *wsp* probe to *Gmm* female DNA restricted with the *Hind*III enzyme produced five bands of about 1200, 1600, 2150, 2600 and 2700 bp (Figure 4, lanes 1 and 3). DNA from tetracycline-treated females (cyt*Wol*-free) had a similar profile, except that the 2700 bp band, corresponding to the expected cyt*Wol wsp* fragment, was absent (lane 2). Untreated male DNA displayed an additional band of 1500 bp, indicating the presence of insertions on the Y chromosome (Lane 4). This banding pattern suggests the presence of at least five independent *wsp* chromosomal insertions, including one on the Y chromosome, supporting the *in sili*co analyses.

### chrWol insertions as determined by FISH

**Figure 5:**
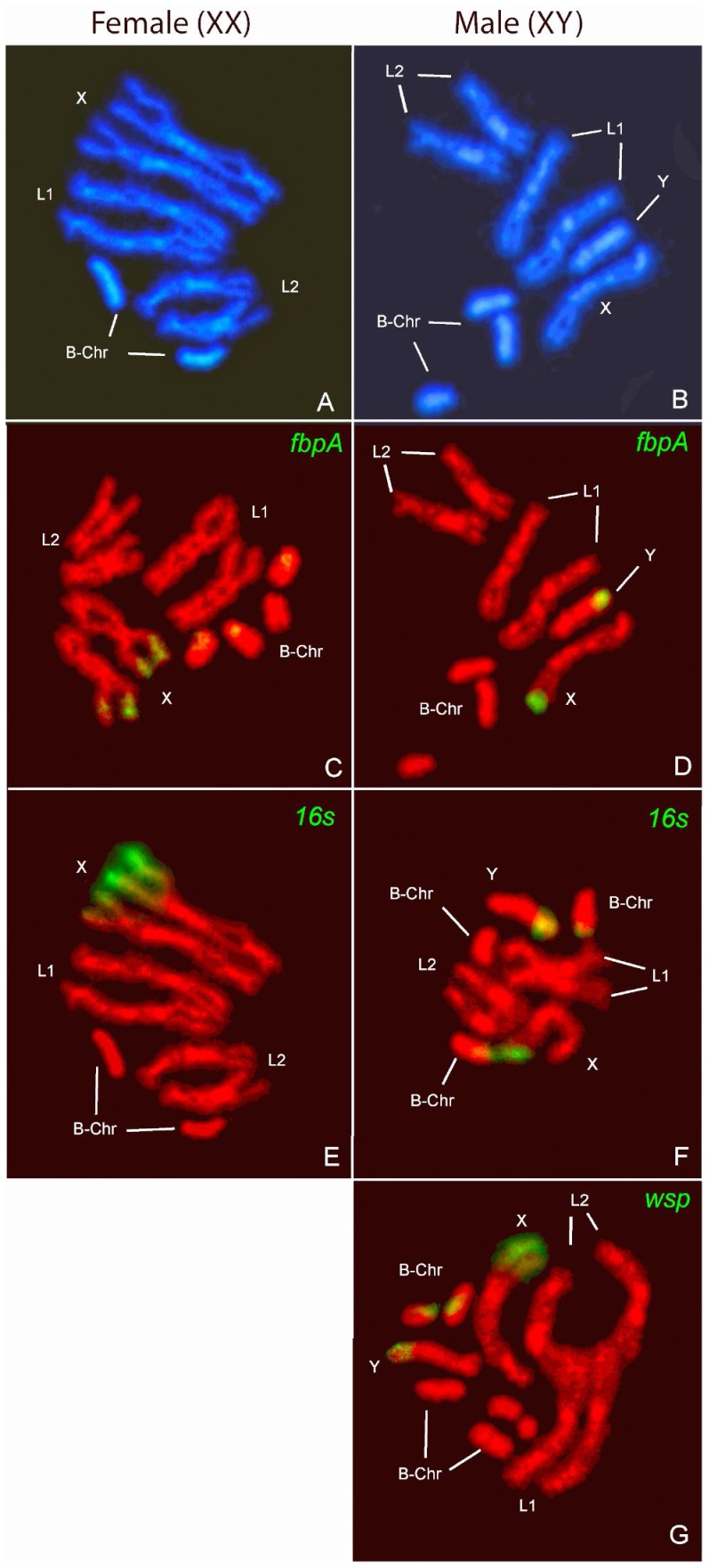
Fluorescent *in situ* hybridisation (FISH) on *Gmm* mitotic chromosomes. The chromosomes are numbered as described by Willhoeft (1997) [70]. A–B. Banding pattern of (DAPI)-stained chromosome spreads (A–B). The DAPI positive regions indicate the heterochromatic patterns. B-chromosomes vary in number. FISH on female and male chromosomes with *fbpA* probe (C–D),*16S rDNA* probe (E–F), and *wsp* probe on chromosomes from a male individual (G).

To determine the location of *Wolbachia* insertions on *Gmm* chromosomes, we performed FISH analyses on mitotic spreads using *wsp*, *16S rRNA* and *fbpA* specific probes. The *Gmm* mitotic complement, comprising the supernumerary dispensable chromosomes (B chr) [78] is depicted in Figure 5, where the AT-rich heterochromatic nature of Y and B chromosomes is indicated by the strong DAPI-staining. The two autosomes, L1 and L2, as well as the X chromosome, appear to contain heterochromatic regions on both sides of the centromere. FISH results indicate that the *Wolbachia* genes *16S*, *fbpA*, and *wsp* consistently display a biased location on the distal part of the X, Y and B chromosomal arm. Although tyramide labeling generates strong and site-specific signals, it is difficult to detect the presence of multiple insertions on one chromosome if these events are localized in close proximity. The *16S* rRNA signal detected on the short arm of the X chromosome appears to be particularly strong and diffused, and may thus represent more than one insertion event in that region.

### HGT events in natural populations of *Gmm*

**Figure 6:**
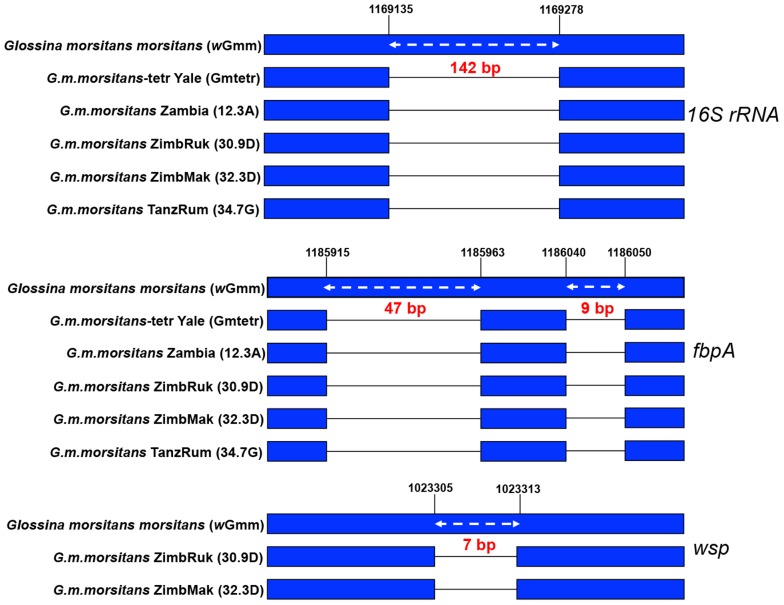
*16S rRNA*, *fbpA* and *wsp* gene fragments from tsetse *Wolbachia* chromosomal insertions sequenced from *Gmm* laboratory and natural populations aligned with the corresponding regions of *w*Gmm. Black lines represent the deleted region. The numbers show the positions before and after the deletions in respect to the *w*Mel genome and the right-left white arrows below the number indicate the size of deletion in base pairs.

Our previous characterization of the laboratory *Gmm* strain by *Wolbachia*-specific *16S rRNA*-based PCR screening, the *wsp*-based and the MLST typing system revealed several HGT events [45]. Our results presented above indicate that these transfer events are in fact more extensive than previously considered. We next investigated the presence of HGT events in natural populations of *Gmm* originating from Zambia, Tanzania and Zimbabwe. We detected the pseudogenized fragment of the *16S rRNA* gene carrying a deletion of 142 bp (Figure 6), similar to that we described in *Gmm* colony DNA prepared from the tetracycline-treated (cyt*Wol*-free) samples [41], [45]. We observed a similar phenomenon for *fbpA*, where a pseudogenized gene fragment could be amplified containing two deletions of 47 and 9 bp from the same four natural populations, as well as from the cyt*Wol*-free *Gmm* laboratory strain DNA sample. Finally, the HGT event of the *Wolbachia wsp* gene, which has been pseudogenized through a deletion of 7 bp, was also detected in two natural samples (Figure 6). Unlike the laboratory line of *Gmm*, in which all individuals analyzed carried the cyt*Wol* strain (100% infected), the prevalence of *Wolbachia* varied in the different populations and was not fixed (Table 1).

## Discussion

Here we report on a newly sequenced genome of cytoplasmic *Wolbachia* strain associated with the tsetse fly *G. m. morsitans.* Previous studies have shown that *w*Gmm belongs to *Wolbachia* supergroup A [79] and functional investigations have demonstrated that this *Wolbachia* strain can induce strong CI in the *Gmm* laboratory line [41]. Our comparative analysis confirms that *w*Gmm belongs to *Wolbachia* supergroup A, and is most similar to *w*Mel, based on the extensive synteny between their genomes. We also show evidence for extensive chromosomal insertions of *w*Gmm in the host genome: with at least two large insertions of 527,507 and 484,123 bp identified from WGS data. Southern blot hybridizations confirmed the presence of *Wolbachia* insertions in the *Gmm* genome, and FISH revealed their biased location on the two sex chromosomes (X and Y), as well as on the supernumerary B-chromosomes.

The genome sequence of the cytoplasmic *w*Gmm strain, when compared to *Wolbachia* genomes from other ecdysozoans, revealed the following striking features: (a) genome size comparable to that of the *w*Bm infecting the filarial nematode *B. malayi*,(b) genome size significantly smaller from all other insect *Wolbachia* strains and particularly from the *w*Pip infecting the mosquito *Culex pipiens*; (c) reduced number of repetitive sequences including ISs, mobile II introns and phages, and (d) absence of functional phage copies. It is worth noting that the genome reduction has not affected the stable symbiotic association, including the expression of strong CI phenomena, as has been documented *in vitro* [41], [80].

Previous research has demonstrated that *Wolbachia* genomes undergo frequent rearrangements and rapid evolution due to the high number of transposable elements and repeat regions, which can provide sites for homologous recombination [18], [81], [82]. The rearrangements in *Wolbachia* may have arisen from the introduction and expansion of the repeat element families that could serve as sites for intragenomic recombination, as has been shown to occur for some other bacterial species [27], [82], [83].

Phylogenetic analysis suggests that the phage of *cyt*Wol (*w*Gmm-WO) and the phage regions present on the two main chromosomal insertions are closely related, implying that the chromosomal phage sequences most likely originated from the cytoplasmic *Wolbachia* phage. However, it appears that the *w*Gmm phage copies are more closely related to the *w*Mel and *w*Ri than the *w*Pip phages. Given that the *Wolbachia* prophages can laterally transfer between *Wolbachia* strains shaping the bacterial genome evolution [84]–[88], the origin of the *w*Gmm phage copies remains an open question.

Of particular interest for host-symbiont interactions are the number of genes that encode proteins that contain ankyrin repeat domains. The ankyrin repeat domain (ANK)-containing proteins, tandem motifs of around 33 amino acids that are involved in protein-protein interactions, are mainly found in eukaryotes and viruses [89]. In eukaryotes, ANK proteins are known to participate in diverse pathways affecting the structure and function of cells regulating host cell cycle or cell division or interacting with the host cytoskeleton [89]–[91]. In addition, they have been shown to act as T4SS effectors participating in host-pathogen interactions [92]. For example, in the intracellular pathogen *Anaplasma phagocytophilum*, AnkA, which is secreted through T4SS, interacts with the host chromatin and regulates gene transcription, while in *Legionella pneumophila*, the AnkX protein prevents microtubule-dependent endocytic maturation of pathogen-occupied vacuoles [92]. While ANK proteins have been reported from bacteria, they are usually present in only a few copies per species [93]. *w*Gmm has 10 putative ANK proteins, comparable to the number reported for other insect *Wolbachia* strains (23 in *w*Mel, 35 in *w*Ri and 60 in *w*Pip). ANK proteins have been considered to play an important role in host-*Wolbachia* interactions, including the establishment of symbiosis. However, their role in the induction of reproductive abnormalities such as CI has not been confirmed as yet [76], [94], [95].

Several studies clearly suggest that the occurrence of HGT events in host-*Wolbachia* symbiotic associations is more widespread than previously thought [18]–[24]. Our results provide evidence of extensive HGT events between *Wolbachia* and tsetse genome, and further advance our knowledge on HGT during their co-evolution. From *in situ* hybridization results, it appears that at least three *Wolbachia* genes, *16S rRNA*, *fbpA*, and *wsp* are located on X, Y and multiple B supernumerary chromosomes. Under the canonical model of sex chromosome evolution, X and Y are believed to have originated from an autosome pair via a three-step process beginning with the acquisition of one or more sex-determining genes [96]–[99]. X and Y are thought to have diverged due to sexually antagonist selection [100], [101]. The suppression of recombination between the two sex chromosomes would be favored by chromosomal inversions and other genetic changes [102]–[105]. As the X became progressively haploid in males (hemizygous), selection may have favored increased transcription of X-linked genes in males through dosage compensation mechanisms [106], [107]. In the later stages, lack of recombination between X and Y allowed for genetic degeneration of the Y, which is usually heterochromatic, accumulating large amounts of repetitive DNA [104], [108], [109]. Due to the highly repetitive nature, the accumulation of *Wolbachia* sequences may not be deleterious for Y functionality, and thus the inserted sequences are not eliminated. The presence of *Wolbachia* HGT events on the B chromosomes may reflect the common evolutionary origin of B and Y chromosomes. Indeed, in *Glossina* species homology between the supernumerary and sex chromosomes has been reported, suggesting the formation of B via Y chromosome duplication and subsequent accumulation of repetitive DNA sequences [110]. However, Carvalho and colleagues (2009) [97] do not exclude the alternative evolutionary scenario of Y originating from B.

The localization of the *Wolbachia* inserts in heterochromatic regions might protect them against the negative selection that would otherwise arise if they were inserted into functional genes, as occurs for transposable elements [111]. However, the heterochromatic location of the insertions may not necessarily imply loss of function, especially for those that are inserted in the facultative heterochromatin. It has been suggested for other insects [23] that *Wolbachia* genes transferred to host chromosomes are structurally disrupted, and functionally impaired via pseudogenization. Through the acquisition of point mutations, insertions and/or deletions, these insertions may be destined to become junk DNA in the insect genome [18]. It has been reported that some horizontally transferred genes can be transcribed in the insect hosts. In the case of the pea aphid *Acyrthosiphon pisum* [112], and the mosquito *Aedes aegypti*, the transferred genes have been found to be transcriptionally active in the salivary glands [18], [19]. In the tripartite mealybug symbiosis, at least twenty-two highly expressed genes have been identified from multiple diverse bacteria [113]. In addition, almost 2% of the *Wolbachia* genes that were transferred to the second chromosome of *D. ananassae* are transcribed [22]. In the case of the nematode *Onchocerca flexuosa*, which does not carry a cytoplasmic *Wolbachia* infection, *Wolbachia*-like DNA sequences have been identified in the nuclear genome [114]. Despite the fact that several of these sequences are degenerate, many are expressed at both the RNA and protein levels [115]. The only case of *Wolbachia* genes transferred to the X chromosome has been reported in the adzuki bean beetle *C. chinensis*, where the insertion was presumably transcriptionally inactive [23], [24]. The present study showed that only a few specific genes may be expressed at low levels from chrWol, however, further studies are required to confirm potential expression of these and or other genes in a temporal and spatial manner. Given the biological interdependence between insect hosts and bacterial symbionts, transfer of symbiont genes of functional (possibly regulatory) relevance may be beneficial for the host. Thus, it is of importance to clarify the potential functional role(s) these inserted sequences may play on host *Gmm* physiology. In addition, whether *Wolbachia* fragments in the *Glossina* genome may be on an evolutionary trajectory of degradation and loss [18] needs to be verified, especially given the large size of the inserts we detected, which may indicate a relatively recent origin for these events.

The origin of horizontal transfer of *Wolbachia* genes in *Gmm* is of evolutionary significance. The phylogenetic analysis presented in Figure S3 shows a long branch from *w*Gmm and short distance between insertion A and insertion B, which strongly support a single transfer event. Also, the genetic distance between several genes present in the cyt*Wol* and their homologues in the chr*Wol* insertions is minimal, thus making it difficult to assess the history of the insertion events. While speculative, it is most likely that the common ancestor for the two chromosomal insertions we detect is the *w*Gmm cytoplasmic strain (Table 5).

It is thought that *Wolbachia* induced CI can promote reproductive isolation in host insects that can potentially lead to speciation [1], [116]. While the genetic mechanism and specific genes involved in CI are currently unknown, if genes involved in CI integrated into the host chromosome were functional, this could result in reproductive isolation and speciation. Unpredictable rates of CI expression could complicate *Wolbachia*-based strategies for tsetse control, if genes involved in the CI mechanism are expressed from chromosomal loci. The results presented here could be used as part of future research to test this hypothesis in tsetse, once the molecular mechanism behind CI has been further defined.

Our analysis with *Gmm* individuals from natural populations indicates the presence of the chromosomal insertions in the field populations as well. Interestingly not all individuals in the field carried the cytoplasmic infections, despite the presence of chromosomal insertions. We can speculate that maternal transmission of *Wolbachia* may be less than perfect in the field, resulting in individuals with no infections. In addition, *Wolbachia* densities have been shown to vary as a function of host age [117], [118], but the field samples could not be scored for relative age. Alternatively, recent studies have identified low-density infections in several tsetse flies including subspecies of *G. morsitans* [45], [116], [119], which could not be detected using the PCR conditions that were employed in this study. Studies that determine infection prevalence or infection densities in natural populations could be compromised if chromosomal sequences are mistaken for cytoplasmic infections. The results raise the question of whether HGT events as shown here are common in other species of tsetse flies, and ongoing WGS of other tsetse species will provide important insights. Future work should focus on determining the prevalence and ancestry of the chromosomal insertions in tsetse.

### List of genes, locus tags and GI numbers

*w*Mel genome (AE017196), *w*Ri genome (CP001391), *w*Pip genome (AM999887), *w*Bm genome (AE017321), DNA-directed RNA polymerase (WD_0024/GI 42409679, WRi_000230/GI:225591874, WP0554/GI:190357240, Wbm0647/GI:58419220), *DNA polymerase III (alpha subunit)* (WD_0780/GI:42410358, WRi_006220/GI:225592374, WP0658/GI:190357336, Wbm0499/GI:58419072), *DNA gyrase B* (WD_0112/GI:42409755, WRi_001420/GI:225591969, WP1103/GI:190357759, Wbm0764/GI:58419337), *translation elongation factor G* (WD_0016/GI:42409671, WRi_000140/GI:225591866, WP0562/GI:190357248, Wbm0344/GI:58418918), *aspartyl-tRNA synthetase* (WD_0413*/*GI:42410026, WRi_003280/GI:225592127, WP0387/GI:190357090, Wbm0012/GI:58418589), *CTP synthase* (WD_0468/GI:42410077, WRi_002850/GI:225592086, WP1235/GI:190357884, Wbm0169/GI:58418745), *glutamyl-tRNA(Gln) amidotransferase B* (WD_0146/GI:42409786, WRi_003090/GI:225592108, WP0087/GI:190356829, Wbm0445/GI:58419018), *GTP-binding protein* (WD_1098/GI:42410645, WRi_012740/GI:225592933, WP0891/GI:190357560, Wbm0032/GI:58418609), *cell division protein FtsZ* (WD_0723/GI:42410305, WRi_007520/GI:225592482, WP0577/GI:190357261, Wbm0602/GI:58419175),fructose-bisphosphate aldolase (WD_1238 GI:42410776, WRi_012130/GI:225592879, WP1081/GI:190357738, Wbm0097/GI:58418674), HK97 family phage major capsid protein (WD_0458/GI:42410067, WRi_002750/GI:225592077, WP0102/GI:190356840), phage integrase family site-specific recombinase (WD_1148/GI:42410690, WRi_009900/GI:225592678, WP0980/GI:190357644), phage SPO1 DNA polymerase-related protein (WD_0164/GI:42409803, WRi_000900/GI:225591926, WP0922/GI:190357589), prophage LambdaW5 baseplate assembly protein W (WD_0640/GI:42410229, WRi_005480/GI:225592306, WP0303/GI:190357018), prophage LambdaW1, baseplate assembly protein J (WD_0639/GI:42410228, WRi_010130/GI:225592699, WP0302/GI:190357017) and a prophage LambdaW1 site-specific recombinase resolvase protein (WD_0634/GI:42410223, WRi_005400/GI:225592300, WP0342/GI:190357056)

## Supporting Information

Figure S1 Maximum Likelihood phylogeny based on phage concatenated genes (5,912 bp). The topology resulting from the Neighbor-Joining method was identical. Strains are characterized by the names of their host species. ML bootstrap values based on 1000 replicates are given.
(TIF)

Figure S2 Circular map of the Type IV genes present in *w*Gmm, *w*Mel, *w*Ri, and *w*Pip. The outermost circle represents the scale in Kbp. In the second circle Type IV genes are colored based on their homology. Regions of homology are connected with bands. Blue ribbons are composed of synteny regions identified using MAUVE and Mummer 3.0 between *w*Mel and *w*Pip. Blue ribbons are composed of synteny regions identified using MAUVE and Mummer 3.0 between *w*Mel and *w*Pip. Light orange ribbons are composed of synteny regions identified using MAUVE and Mummer 3.0 between *w*Mel and *w*Ri. Light grey ribbons are composed of synteny regions, identified using Mauve and Mummer 3.0, between *w*Mel and *w*Gmm.
(TIF)

Figure S3 Maximum Likelihood phylogeny based on ten concatenated genes (25,578 bp). The topology resulting from the Neighbor-Joining method was identical. Strains are characterized by the names of their host species. ML bootstrap values based on 1000 replicates are given.
(TIF)

Figure S4 LASTZ identity plots based on genomic pairwise alignment between (A) wGmm and insertion A, (B) wGmm and insertion B, and (C) insertion A and insertion B. For plots (A) and (B) the size of the *w*Gmm genome is given in x-axis, while for plot (C) the size of insertion A. High-scoring segment pairs are presented as blue dots while low-scoring segment pairs are presented as red dots.
(TIF)

Table S1 Assembly statistics after each major stage of the assembly process of *w*Gmm draft genome.
(DOCX)

Table S2 Number of unique genes present in *w*Gmm compared with the genomes of *w*Mel, *w*Ri, *w*Pip and *w*Bm.
(DOCX)

Table S3 Missing regions and genes from the *w*Gmm genome in respect to *w*Mel. Regions have been identified after alignment of the two genomes with MAUVE using the default settings of the program. Gaps in the genomes were identified using Geneious.
(DOCX)

Table S4 Missing regions and genes from the *w*Gmm genome in respect to *w*Ri. Alignment of the two genomes was performed with MAUVE using the default settings of the program. Gaps in the genomes were identified using Geneious v. 5.4.
(DOCX)

Table S5 Features and comparisons of the *w*Gmm genome and chromosomal insertions with other sequenced *Wolbachia* genomes. Alignment of the two genomes was performed with MAUVE using the default settings of the program. Gaps in the genomes were identified using Geneious v. 5.4.
(DOCX)

Table S6 Description of the first set of *Wolbachia* inserted regions into the *G. m. morsitans* chromosomes.
(DOCX)

Table S7 Description of the second set of *Wolbachia* inserted regions into the *G. m. morsitans* chromosomes.
(DOCX)

Table S8 Description of the third set of *Wolbachia* inserted regions into the *G. m. morsitans* chromosomes.
(DOCX)
