# Supplementary figures and images for: Presence of Extensive Wolbachia Symbiont Insertions Discovered in the Genome of Its Host Glossina morsitans morsitans
Source: PLoS Negl Trop Dis. 2014 Apr 24;8(4):e2728. doi: 10.1371/journal.pntd.0002728 (PMC3998919; doi:10.1371/journal.pntd.0002728)

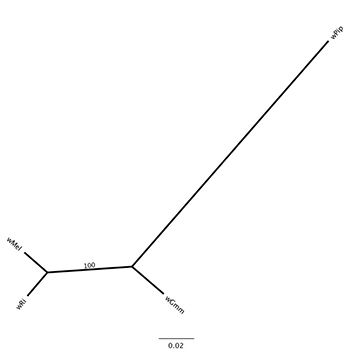

Supplement: Figure S1 — Maximum Likelihood phylogeny based on phage concatenated genes (5,912 bp). The topology resulting from the Neighbor-Joining method was identical. Strains are characterized by the names of their host species. ML bootstrap values based on 1000 replicates are given. (TIF) [file pntd.0002728.s001.tif]

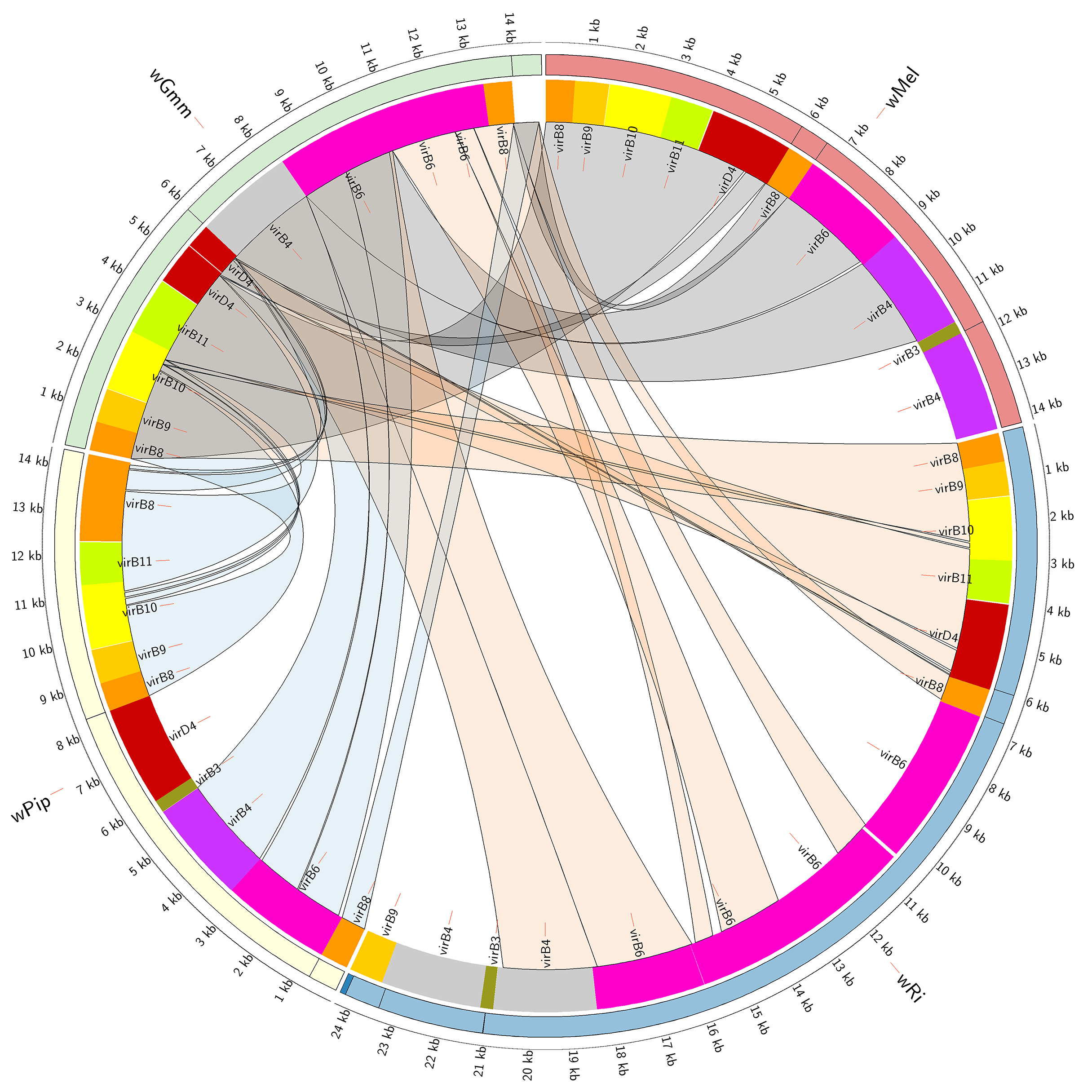

Supplement: Figure S2 — Circular map of the Type IV genes present in wGmm, wMel, wRi, and wPip. The outermost circle represents the scale in Kbp. In the second circle Type IV genes are colored based on their homology. Regions of homology are connected with bands. Blue ribbons are composed of synteny regions identified using MAUVE and Mummer 3.0 between wMel and wPip. Blue ribbons are composed of synteny regions identified using MAUVE and Mummer 3.0 between wMel and wPip. Light orange ribbons are composed of synteny regions identified using MAUVE and Mummer 3.0 between wMel and wRi. Light grey ribbons are composed of synteny regions, identified using Mauve and Mummer 3.0, between wMel and wGmm. (TIF) [file pntd.0002728.s002.tif]

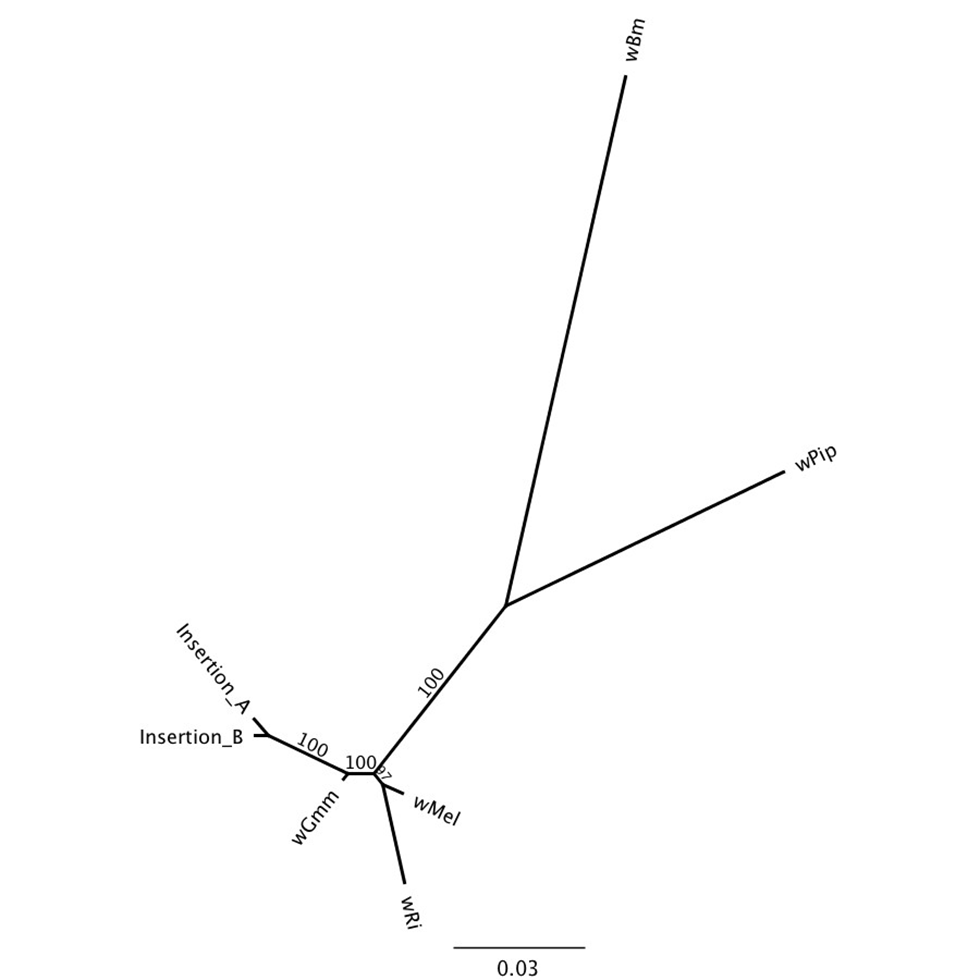

Supplement: Figure S3 — Maximum Likelihood phylogeny based on ten concatenated genes (25,578 bp). The topology resulting from the Neighbor-Joining method was identical. Strains are characterized by the names of their host species. ML bootstrap values based on 1000 replicates are given. (TIF) [file pntd.0002728.s003.tif]

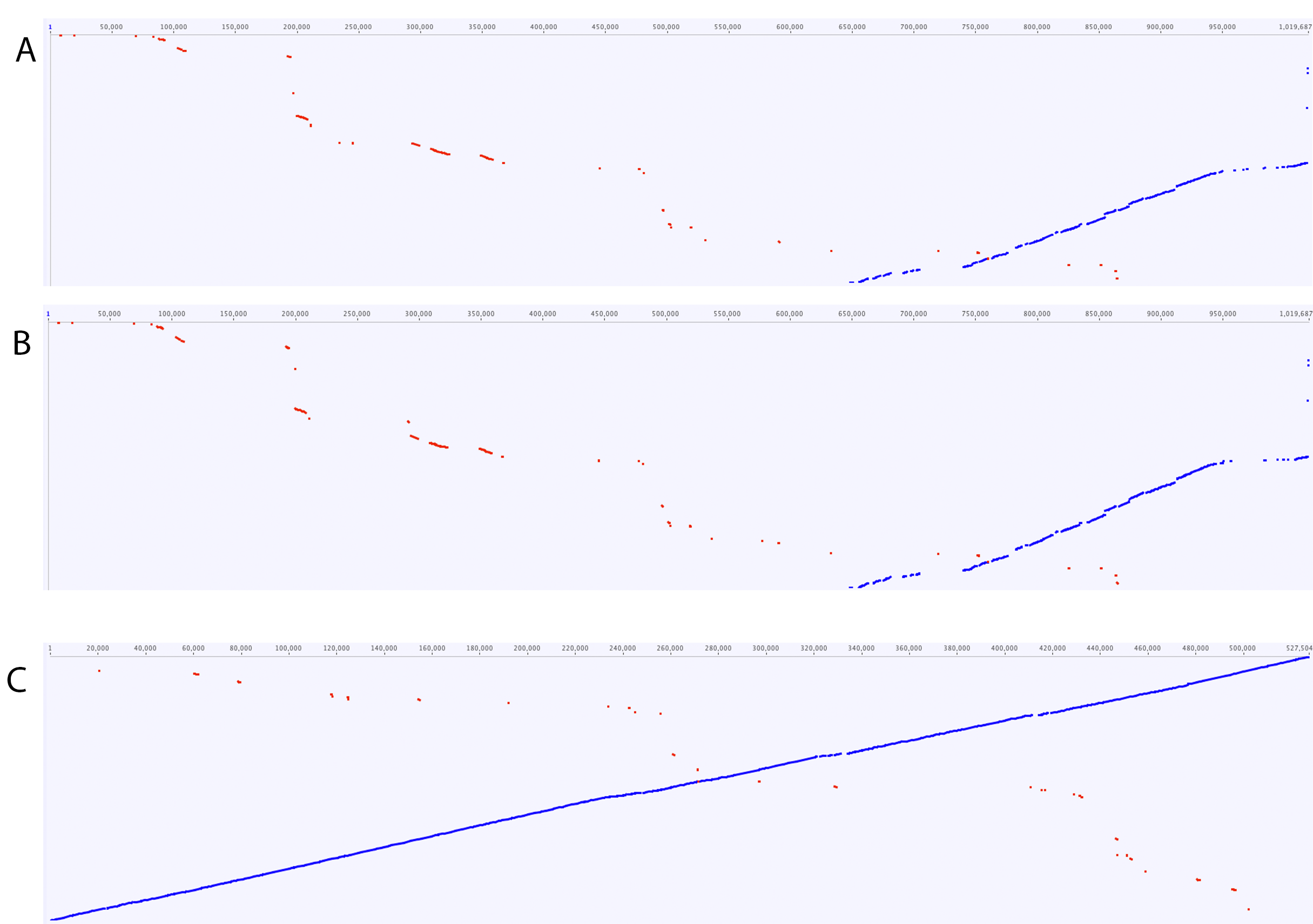

Supplement: Figure S4 — LASTZ identity plots based on genomic pairwise alignment between (A) wGmm and insertion A, (B) wGmm and insertion B, and (C) insertion A and insertion B. For plots (A) and (B) the size of the wGmm genome is given in x-axis, while for plot (C) the size of insertion A. High-scoring segment pairs are presented as blue dots while low-scoring segment pairs are presented as red dots. (TIF) [file pntd.0002728.s004.tif]
