# Supplementary material for: Presence of Extensive Wolbachia Symbiont Insertions Discovered in the Genome of Its Host Glossina morsitans morsitans
Source: PLoS Negl Trop Dis. 2014 Apr 24;8(4):e2728. doi: 10.1371/journal.pntd.0002728 (PMC3998919; doi:10.1371/journal.pntd.0002728)
Supplement: Table S1 — Assembly statistics after each major stage of the assembly process of wGmm draft genome. (DOCX) [file pntd.0002728.s005.docx]

**Table S1.** Assembly statistics after each major stage of the assembly process of *w*Gmm draft genome.

|  | Total length (bp) | Number of sequences | N50 statistic (bp) | Mean length (bp) | Longest contig (bp) |
| --- | --- | --- | --- | --- | --- |
| Initial assembly | 883176 | 291 | 3897 | 3034 | 15356 |
| IMAGE gap closing | 1020356 | 231 | 6258 | 4417 | 27995 |
| SCARPA scaffolding | 1020085 | 201 | 8172 | 5075 | 27995 |
