# Supplementary material for: Presence of Extensive Wolbachia Symbiont Insertions Discovered in the Genome of Its Host Glossina morsitans morsitans
Source: PLoS Negl Trop Dis. 2014 Apr 24;8(4):e2728. doi: 10.1371/journal.pntd.0002728 (PMC3998919; doi:10.1371/journal.pntd.0002728)
Supplement: Table S2 — Number of unique genes present in wGmm compared with the genomes of wMel, wRi, wPip and wBm. (DOCX) [file pntd.0002728.s006.docx]

**Table S2.** Number of unique genes present in *w*Gmm compared with the genomes of *w*Mel, *w*Ri, *w*Pip and *w*Bm.

|  | *w*Mel | *w*Ri | *w*Pip | *w*Bm | *w*Mel, *w*Ri, *w*Pip, *w*Bm |
| --- | --- | --- | --- | --- | --- |
| *w*Gmm | 1 | 6 | 29 | 36 | 0 |
