# Supplementary material for: Presence of Extensive Wolbachia Symbiont Insertions Discovered in the Genome of Its Host Glossina morsitans morsitans
Source: PLoS Negl Trop Dis. 2014 Apr 24;8(4):e2728. doi: 10.1371/journal.pntd.0002728 (PMC3998919; doi:10.1371/journal.pntd.0002728)
Supplement: Table S3 — Missing regions and genes from the wGmm genome in respect to wMel. Regions have been identified after alignment of the two genomes with MAUVE using the default settings of the program. Gaps in the genomes were identified using Geneious. (DOCX) [file pntd.0002728.s007.docx]

**Table S3.** Missing regions and genes from the *w*Gmm genome in respect to *w*Mel. Regions have been identified after alignment of the two genomes with MAUVE using the default settings of the program. Gaps in the genomes were identified using Geneious.

| Missing Region 1 (240000-250000) | Locus Tag |
| --- | --- |
| transposase, IS4 family CDS | WD_0252 |
| transposase, IS256 family CDS | WD_0253 |
| transcriptional regulator, putative CDS | WD_0254 |
| transcriptional regulator, putative CDS | WD_0255 |
| hypothetical protein CDS | WD_0256 |
| DNA repair protein RadC, truncation CDS | WD_0257 |
| conserved hypothetical protein CDS | WD_0259 |
| conserved hypothetical protein, interruption-N CDS | WD_0261 |
| conserved hypothetical protein, interruption-C CDS | WD_0262 |
| prophage LambdaW1, DNA methylase CDS | WD_0263 |
| conserved hypothetical protein CDS | WD_0264 |
| Missing Region 2 (265000-276000) |  |
| ankyrin repeat domain protein CDS | WD_0294 |
| conserved hypothetical protein CDS | WD_0279 |
| conserved hypothetical protein CDS | WD_0284 |
| hypothetical protein CDS | WD_0289 |
| hypothetical protein CDS | WD_0290 |
| hypothetical protein CDS | WD_0293 |
| hypothetical protein CDS | WD_0295 |
| prophage LambdaW1, ankyrin repeat domain protein CDS | WD_0285 |
| prophage LambdaW1, ankyrin repeat domain protein CDS | WD_286 |
| prophage LambdaW1, ankyrin repeat domain protein CDS | WD_0291 |
| prophage LambdaW1, ankyrin repeat domain protein CDS | WD_0292 |
| prophage LambdaW1, site-specific recombinase, resolvase family CDS | WD_0288 |
| Missing Region 3 (486000-510000) |  |
| DNA repair protein RadC, truncation CDS | WD_0507 |
| transcriptional regulator, putative CDS | WD_0508 |
| mutL CDS | WD_0509 |
| conserved hypothetical protein CDS | WD_0511 |
| hypothetical protein CDS | WD_0512 |
| hypothetical protein CDS | WD_0513 |
| ankyrin repeat domain protein CDS | WD_0514 |
| reverse transcriptase, interruption-C CDS | WD_0515 |
| transposase, IS5 family, OrfB CDS | WD_0516 |
| transposase, IS5 family, OrfA CDS | WD_0517 |
|  |  |
|  |  |
|  |  |
|  |  |
|  |  |
|  |  |
|  |  |
| Missing Region 4 (549000-590000) |  |
| transposase, IS4 family CDS | WD_0563 |
| hypothetical protein CDS | WD_0564 |
| patatin family protein CDS | WD_0565 |
| ankyrin repeat domain protein CDS | WD_0566 |
| prophage P2W3, tail protein D, putative CDS | WD_0567 |
| prophage P2W3, tail protein X, putative CDS | WD_0568 |
| prophage P2W3, tail protein U, putative CDS | WD_0569 |
| prophage P2W3, tail tape measure protein CDS | WD_0570 |
| hypothetical protein CDS | WD_0571 |
| conserved hypothetical protein CDS | WD_0572 |
| hypothetical protein CDS | WD_0573 |
| prophage P2W3, contractile tail tube protein CDS | WD_0574 |
| hypothetical protein CDS | WD_0576 |
| hypothetical protein CDS | WD_0577 |
| hypothetical protein CDS | WD_0578 |
| hypothetical protein CDS | WD_0579 |
| hypothetical protein CDS | WD_0580 |
| hypothetical protein CDS | WD_0581 |
| regulatory protein RepA, putative CDS | WD_0582 |
| hypothetical protein CDS | WD_0583 |
| hypothetical protein CDS | WD_0584 |
| conserved hypothetical protein CDS | WD_0585 |
| hypothetical protein CDS | WD_0586 |
| transposase, IS5 family, OrfB CDS | WD_0587 |
| transposase, IS5 family, OrfA CDS | WD_0588 |
| hypothetical protein CDS | WD_0589 |
| conserved hypothetical protein CDS | WD_0590 |
| conserved hypothetical protein CDS | WD_0591 |
| prophage LambdaW4, DNA methylase CDS | WD_0594 |
| conserved hypothetical protein CDS | WD_0595 |
| prophage LambdaW4, ankyrin repeat domain protein CDS | WD_0596 |
| prophage LambdaW4, terminase large subunit, putative CDS | WD_0597 |
| hypothetical protein CDS | WD_0598 |
| hypothetical protein CDS | WD_0599 |
| conserved hypothetical protein CDS | WD_0600 |
| prophage LambdaW4, minor capsid protein C, putative CDS | WD_0602 |
| conserved hypothetical protein CDS | WD_0603 |
| conserved hypothetical protein CDS | WD_0604 |
| hypothetical protein CDS | WD_0605 |
| hypothetical protein CDS | WD_0607 |
| hypothetical protein CDS | WD_0608 |
| regulatory protein RepA, putative CDS | WD_0609 |
| helicase, SNF2 family CDS | WD_0610 |
| UDP-N-acetylglucosamine pyrophosphorylase-related protein CDS | WD_0611 |
| NAD-dependent epimerase/dehydratase family protein CDS | WD_0612 |
| glycosyl transferase, group 1 family protein / moaA/nifB/pqqE family protein CDS | WD_0613 |
| hypothetical protein CDS | WD_0614 |
| conserved domain protein CDS | WD_0615 |
| ABC transporter, permease/ATP-binding protein, putative CDS | WD_0616 |
| L-allo-threonine aldolase, putative CDS | WD_0617 |
| L-allo-threonine aldolase, putative CDS | WD_0618 |
| GlpT/PgpT/UhpT transporter family protein CDS | WD_0619 |
| UDP-glucose 6-dehydrogenase CDS | WD_0620 |
| membrane protein, putative CDS | WD_0621 |
| transcriptional regulator, putative CDS | WD_0622 |
| transcriptional regulator, putative CDS | WD_0623 |
| DNA repair protein RadC, putative CDS | WD_0625 |
| transcriptional regulator, putative CDS | WD_0626 |
| conserved hypothetical protein CDS | WD_0627 |
| hypothetical protein CDS | WD_0628 |
| hypothetical protein CDS | WD_0630 |
| hypothetical protein CDS | WD_0631 |
| misc |  |
| misc |  |
