# Supplementary material for: Presence of Extensive Wolbachia Symbiont Insertions Discovered in the Genome of Its Host Glossina morsitans morsitans
Source: PLoS Negl Trop Dis. 2014 Apr 24;8(4):e2728. doi: 10.1371/journal.pntd.0002728 (PMC3998919; doi:10.1371/journal.pntd.0002728)
Supplement: Table S4 — Missing regions and genes from the wGmm genome in respect to wRi. Alignment of the two genomes was performed with MAUVE using the default settings of the program. Gaps in the genomes were identified using Geneious v. 5.4. (DOCX) [file pntd.0002728.s008.docx]

**Table S4.** Missing regions and genes from the *w*Gmm genome in respect to *w*Ri. Alignment of the two genomes was performed with MAUVE using the default settings of the program. Gaps in the genomes were identified using Geneious v. 5.4.

| Missing_region_1 (561000-640000) | Locus_tag |
| --- | --- |
| ABC transporter, permease/ATP-binding protein CDS | WRi_005780 |
| ankyrin repeat domain protein CDS | WRi_005390 |
| ankyrin repeat domain protein CDS | WRi_005440 |
| ankyrin repeat domain protein CDS | WRi_005450 |
| ankyrin repeat domain protein CDS | WRi_005620 |
| baseplate assembly protein J, putative CDS | WRi_005470 |
| baseplate assembly protein V CDS | WRi_005500 |
| baseplate assembly protein W, putative CDS | WRi_005480 |
| DNA methylase CDS | WRi_005640 |
| DNA repair protein RadC, putative CDS | WRi_005870 |
| glycosyl transferase, group 1 family protein / moaA/nifB/pqqE family protein CDS | WRi_005750 |
| helicase, SNF2 family CDS | WRi_005720 |
| hypothetical protein CDS | WRi_005310 |
| hypothetical protein CDS | WRi_005320 |
| hypothetical protein CDS | WRi_005340 |
| hypothetical protein CDS | WRi_005350 |
| hypothetical protein CDS | WRi_005360 |
| hypothetical protein CDS | WRi_005370 |
| hypothetical protein CDS | WRi_005460 |
| hypothetical protein CDS | WRi_005490 |
| hypothetical protein CDS | WRi_005510 |
| hypothetical protein CDS | WRi_005530 |
| hypothetical protein CDS | WRi_005540 |
| hypothetical protein CDS | WRi_005550 |
| hypothetical protein CDS | WRi_005590 |
| hypothetical protein CDS | WRi_005600 |
| hypothetical protein CDS | WRi_005630 |
| hypothetical protein CDS | WRi_005660 |
| hypothetical protein CDS | WRi_005680 |
| hypothetical protein CDS | WRi_005690 |
| hypothetical protein CDS | WRi_005760 |
| hypothetical protein CDS | WRi_005770 |
| hypothetical protein CDS | WRi_005830 |
| hypothetical protein CDS | WRi_005860 |
| hypothetical protein CDS | WRi_005890 |
| hypothetical protein CDS | WRi_005900 |
| hypothetical protein CDS | WRi_005930 |
| L-allo-threonine aldolase, putative CDS | WRi_005790 |
| L-allo-threonine aldolase, putative CDS | WRi_005800 |
| Major facilitator family transporter CDS | WRi_005810 |
| minor capsid protein C, putative CDS | WRi_005560 |
| minor tail protein Z, putative CDS | WRi_005520 |
| NAD-dependent epimerase/dehydratase family protein CDS | WRi_005740 |
| portal protein, lambda family CDS | WRi_005570 |
| ppdK CDS | WRi_005300 |
| Putative addiction module toxin protein CDS | WRi_005580 |
| Putative Holliday junction resolvasome, endonuclease subunit CDS | WRi_005650 |
| putative phage terminase large subunit CDS | WRi_005610 |
| regulatory protein RepA, putative CDS | WRi_005700 |
| reverse transcriptase, putative CDS | WRi_005330 |
| RNA-directed DNA polymerase (Reverse transcriptase) CDS | WRi_005670 |
| site-specific recombinase, resolvase family CDS | WRi_005400 |
| transcriptional regulator, putative CDS | WRi_005840 |
| transcriptional regulator, putative CDS | WRi_005850 |
| transcriptional regulator, putative CDS | WRi_005880 |
| transposase CDS | WRi_005420 |
| transposase CDS | WRi_005710 |
| transposase CDS | WRi_005920 |
| transposase CDS | WRi_005940 |
| UDP-glucose 6-dehydrogenase CDS | WRi_005820 |
| UDP-N-acetylglucosamine pyrophosphorylase-related protein CDS | WRi_005730 |
| Missing_region_2 (715000-771000) |  |
| hypothetical protein CDS | WRi_006640 |
| hypothetical protein CDS | WRi_006650 |
| radC CDS | WRi_006620 |
| Ankyrin repeat domain protein CDS | WRi_006740 |
| Ankyrin repeat domain protein CDS | WRi_006750 |
| ankyrin repeat domain protein CDS | WRi_006810 |
| ankyrin repeat domain protein CDS | WRi_006860 |
| ankyrin repeat domain protein CDS | WRi_006870 |
| ankyrin repeat domain protein CDS | WRi_006900 |
| baseplate assembly protein J, putative CDS | WRi_007080 |
| baseplate assembly protein W, putative CDS | WRi_007090 |
| contractile tail tube protein CDS | WRi_006970 |
| hspC2 CDS | WRi_006800 |
| hypothetical protein CDS | WRi_006790 |
| hypothetical protein CDS | WRi_006890 |
| hypothetical protein CDS | WRi_006950 |
| hypothetical protein CDS | WRi_006960 |
| hypothetical protein CDS | WRi_006990 |
| hypothetical protein CDS | WRi_007000 |
| hypothetical protein CDS | WRi_007010 |
| hypothetical protein CDS | WRi_007020 |
| hypothetical protein CDS | WRi_007060 |
| hypothetical protein CDS | WRi_007070 |
| patatin family protein CDS | WRi_006880 |
| phage tail sheath protein CDS | WRi_006980 |
| tail protein D, putative CDS | WRi_006910 |
| tail protein U, putative CDS | WRi_006930 |
| tail protein X, putative CDS | WRi_006920 |
| tail tape measure protein CDS | WRi_006940 |
| transposase CDS | WRi_006760 |
| transposase CDS | WRi_006770 |
| transposase, IS5 family CDS | WRi_006820 |
| transposase, IS5 family CDS | WRi_007040 |
| Missing_region_3 (772000-784000) |  |
| ankyrin repeat domain protein CDS | WRi_007240 |
| hypothetical protein CDS | WRi_007120 |
| hypothetical protein CDS | WRi_007140 |
| hypothetical protein CDS | WRi_007150 |
| hypothetical protein CDS | WRi_007160 |
| hypothetical protein CDS | WRi_007200 |
| minor capsid protein C, putative CDS | WRi_007170 |
| minor tail protein Z, putative CDS | WRi_007130 |
| phage uncharacterized protein CDS | WRi_007190 |
| portal protein, lambda family CDS | WRi_007180 |
| site-specific recombinase, phage integrase family CDS | WRi_007250 |
| terminase large subunit, putative CDS | WRi_007210 |
| Missing_region_4 (817000-830000) |  |
| hypothetical protein CDS | WRi_007560 |
| hypothetical protein CDS | WRi_007570 |
| Mg chelatase-related protein CDS | WRi_007540 |
| Putative cassette chromosome recombinase resolvase, CcrB-like protein CDS | WRi_007550 |
| regulatory protein RepA, putative CDS | WRi_007580 |
| hypothetical protein CDS | WRi_007590 |
| hypothetical protein CDS | WRi_007600 |
| hypothetical protein CDS | WRi_007610 |
| hypothetical protein CDS | WRi_007620 |
| hypothetical protein CDS | WRi_007630 |
| hypothetical protein CDS | WRi_007640 |
| regulatory protein RepA, putative CDS | WRi_007580 |
| transposase CDS | WRi_007660 |
| Missing_region_5 (1070000-1141000) |  |
| ABC transporter, permease/ATP-binding protein CDS | WRi_0010440 |
| ankyrin repeat domain protein CDS | WRi_0010050 |
| ankyrin repeat domain protein CDS | WRi_0010010 |
| ankyrin repeat domain protein CDS | WRi_0010110 |
| ankyrin repeat domain protein CDS | WRi_0010280 |
| baseplate assembly protein J, putative CDS | WRi_0010130 |
| baseplate assembly protein V CDS | WRi_0010160 |
| baseplate assembly protein W, putative CDS | WRi_0010140 |
| DNA methylase CDS | WRi_0010300 |
| DNA repair protein RadC, putative CDS | WRi_0010530 |
| glycosyl transferase, group 1 / radical SAM family protein CDS | WRi_0010410 |
| helicase, SNF2 family CDS | WRi_0010380 |
| Holliday junction resolvasome, endonuclease subunit CDS | WRi_0010310 |
| hypothetical protein CDS | WRi_009980 |
| hypothetical protein CDS | WRi_009985 |
| hypothetical protein CDS | WRi_010000 |
| hypothetical protein CDS | WRi_010010 |
| hypothetical protein CDS | WRi_010020 |
| hypothetical protein CDS | WRi_010030 |
| hypothetical protein CDS | WRi_010120 |
| hypothetical protein CDS | WRi_010150 |
| hypothetical protein CDS | WRi_010170 |
| hypothetical protein CDS | WRi_010190 |
| hypothetical protein CDS | WRi_010200 |
| hypothetical protein CDS | WRi_010210 |
| hypothetical protein CDS | WRi_010250 |
| hypothetical protein CDS | WRi_010260 |
| hypothetical protein CDS | WRi_010290 |
| hypothetical protein CDS | WRi_010320 |
| hypothetical protein CDS | WRi_010340 |
| hypothetical protein CDS | WRi_010350 |
| hypothetical protein CDS | WRi_010420 |
| hypothetical protein CDS | WRi_010430 |
| hypothetical protein CDS | WRi_010490 |
| hypothetical protein CDS | WRi_010520 |
| hypothetical protein CDS | WRi_010550 |
| hypothetical protein CDS | WRi_010560 |
| L-allo-threonine aldolase, putative CDS | WRi_0010540 |
| L-allo-threonine aldolase, putative CDS | WRi_0010450 |
| Major facilitator family transporter CDS | WRi_0010470 |
| minor capsid protein C, putative CDS | WRi_0010220 |
| minor tail protein Z, putative CDS | WRi_0010180 |
| NAD-dependent epimerase/dehydratase family protein CDS | WRi_0010400 |
| prophage portal protein, lambda family CDS | WRi_0010230 |
| Putative addiction module toxin protein CDS | WRi_0010240 |
| putative phage terminase large subunit CDS | WRi_0010270 |
| regulatory protein RepA, putative CDS | WRi_0010360 |
| reverse transcriptase, putative CDS | WRi_0010570 |
| RNA-directed DNA polymerase (Reverse transcriptase) CDS | WRi_0010330 |
| site-specific recombinase, resolvase family CDS | WRi_0010060 |
| transcriptional regulator, putative CDS | WRi_0010500 |
| transcriptional regulator, putative CDS | WRi_0010510 |
| transcriptional regulator, putative CDS | WRi_0010540 |
| transposase CDS | WRi_0010370 |
| transposase, IS5 family CDS | WRi_0010080 |
| UDP-glucose 6-dehydrogenase CDS | WRi_0010480 |
| UDP-N-acetylglucosamine pyrophosphorylase-related protein CDS | WRi_0010390 |
