# Supplementary material for: Presence of Extensive Wolbachia Symbiont Insertions Discovered in the Genome of Its Host Glossina morsitans morsitans
Source: PLoS Negl Trop Dis. 2014 Apr 24;8(4):e2728. doi: 10.1371/journal.pntd.0002728 (PMC3998919; doi:10.1371/journal.pntd.0002728)
Supplement: Table S5 — Features and comparisons of the wGmm genome and chromosomal insertions with other sequenced Wolbachia genomes. Alignment of the two genomes was performed with MAUVE using the default settings of the program. Gaps in the genomes were identified using Geneious v. 5.4. (DOCX) [file pntd.0002728.s009.docx]

**Table S5.** Features and comparisons of the *w*Gmm genome and chromosomal insertions with other sequenced *Wolbachia* genomes. Alignment of the two genomes was performed with MAUVE using the default settings of the program. Gaps in the genomes were identified using Geneious v. 5.4.

|  | ***w*Mel** | ***w*Ri** | ***w*Gmm** | **Insertion A** | **Insertion B** | **Insertion C** |
| --- | --- | --- | --- | --- | --- | --- |
| **Genome size** | 1,267,782 | 1,445,873 | 1,020,085 | 527,507 | 484,123 | 2,089 |
| **Predicted CDS** | 1,195 | 1,150 | 801 | 197 | 159 | 0 |
| **Pseudogenes** | 113 (8.9%) | 114 (9.9%) | 106  (13.2%) | 148 (42.9%) | 148  (48.2%) | 0 |
| **Gene remnants** | - | - | - | 163 | 157 | 6 |
| **Percent coding** | 85.1% | 80% | 64.7% | 53.6% | 49.9% | 0 |
| **Average length of CDS** | 851 | 976 | 823 | 677 | 690 | n/a |
| **Hypothetical** | 337 (26.5%) | 320 (27.8%) | 218  (27.2%) | 116  (22.05%) | 104  (22.03%) | 0 |
| **Transfer RNA** | 34 | 34 | 34 | 15 | 13 | 0 |
| **GC content** | 35.2% | 35.2 | 35.2% | 35.1% | 35.1% | 34.8% |
