# Supplementary material for: Presence of Extensive Wolbachia Symbiont Insertions Discovered in the Genome of Its Host Glossina morsitans morsitans
Source: PLoS Negl Trop Dis. 2014 Apr 24;8(4):e2728. doi: 10.1371/journal.pntd.0002728 (PMC3998919; doi:10.1371/journal.pntd.0002728)
Supplement: Table S6 — Description of the first set of Wolbachia inserted regions into the G. m. morsitans chromosomes. (DOCX) [file pntd.0002728.s010.docx]

**Table S6.** Description of the first set of *Wolbachia* inserted regions (Insertion A) into the *G. m. morsitans* chromosomes

| **Product** | **Homolog** | **Type** | **Insertion** | **Length** |
| --- | --- | --- | --- | --- |
| (3R)-hydroxymyristoyl-ACP dehydratase | WD1083 | coding | Full | 432 |
| 1-acyl-sn-glycerol-3-phosphate acyltransferase family protein | WRi_009890 | Non-coding | Partial | 601 |
| 1-deoxy-D-xylulose 5-phosphate reductoisomerase | WD0992 | Non-coding | Full | 1160 |
| 2,3,4,5-tetrahydropyridine-2,6-carboxylate N- succinyltransferase | WD0714 | coding | Full | 831 |
| 23S ribosomal RNA | WRi_r01850 | rRNA | rRNA | 2373 |
| 2-oxoglutarate dehydrogenase, E2 component, dihydrolipoamide succinyltransferase | WD0544 | coding | Full | 1173 |
| 3,4-dihydroxy-2-butanone 4-phosphate synthase, putative | WD0653 | coding | Full | 396 |
| 30S ribosomal protein S1 | WD1090 | Non-coding | Partial | 1294 |
| 30S ribosomal protein S1 | WD1090 | Non-coding | Partial | 386 |
| 30S ribosomal protein S10 | WD0682 | Non-coding | Full | 320 |
| 30S ribosomal protein S11 | WD0659 | Non-coding | Full | 448 |
| 30S ribosomal protein S13 | WD0660 | Non-coding | Full | 133 |
| 30S ribosomal protein S16 | WD0798 | coding | Full | 321 |
| 30S ribosomal protein S19 | WD0677 | Non-coding | Full | 285 |
| 30S ribosomal protein S2 | WD0532 | non-coding | Partial | 342 |
| 30S ribosomal protein S4 | WD0388 | non-coding | Partial | 334 |
| 30S ribosomal protein S4 | WD0388 | non-coding | Partial | 153 |
| 30S ribosomal protein S6 | WD0781 | coding | Full | 597 |
| 3-demethylubiquinone-9 3-methyltransferase | WRi_004370 | non-coding | Partial | 532 |
| 3-oxoacyl-(acyl-carrier-protein) reductase | WD0650 | Non-coding | Full | 735 |
| 3-oxoacyl-(acyl-carrier-protein) synthase II | WD1194 | coding | Full | 1267 |
| 4-diphosphocytidyl-2C-methyl-D-erythritol kinase | WD0360 | coding | Full | 645 |
| 4-hydroxy-3-methylbut-2-en-1-yl diphosphate synthase | WD0116 | non-coding | Partial | 1294 |
| 4-hydroxybenzoate polyprenyltransferase | WD1316 | Non-coding | Partial | 695 |
| 50S ribosomal protein L1 | WD0657 | coding | Full | 654 |
| 50S ribosomal protein L10 | WD0022 | coding | Full | 511 |
| 50S ribosomal protein L11 | WD0020 | coding | Full | 267 |
| 50S ribosomal protein L17 | WD0657 | Non-coding | Full | 429 |
| 50S ribosomal protein L2 | WD0391 | coding | Full | 825 |
| 50S ribosomal protein L20 | WD0865 | coding | Full | 315 |
| 50S ribosomal protein L22 | WD0676 | non-coding | Partial | 305 |
| 50S ribosomal protein L23 | WD0679 | coding | Full | 288 |
| 50S ribosomal protein L28 | WD0391 | coding | Full | 189 |
| 50S ribosomal protein L3 | WD0681 | Non-coding | Full | 725 |
| 50S ribosomal protein L4 | WD0680 | Non-coding | Full | 604 |
| 50S ribosomal protein L7/L12 | WD0023 | coding | Full | 404 |
| 50S ribosomal protein L9 | WD0783 | coding | Full | 555 |
| 5S ribosomal RNA | WRi_r01860 | rRNA | rRNA | 111 |
| AAA family ATPase | WD0472 | Non- coding | Full | 1093 |
| ABC transporter, ATP-binding protein | WD0707 | Non- coding | Full | 704 |
| ABC transporter, ATP-binding protein | WD0990 | Non- coding | Full | 478 |
| ABC transporter, periplasmic substrate-binding protein, putative | WD1105 | coding | Full | 456 |
| acetylornithine transaminase protein | WD0559 | Non-coding | Full | 1180 |
| acetyltransferase | WD0729 | coding | Full | 807 |
| aconitate hydratase | WD0105 | Non-coding | Full | 2411 |
| acyl carrier protein | WD1193 | coding | Full | 261 |
| adenylate kinase | WD0661 | Non-coding | Full | 486 |
| adenylosuccinate lyase | WD0786 | coding | Full | 1290 |
| adenylosuccinate synthetase | WD0337 | coding | Full | 1272 |
| alanyl-tRNA synthetase | WD_0862 | non-coding | Partial | 241 |
| alpha/beta fold family hydrolase | WD0802 | non-coding | Partial | 412 |
| alpha/beta fold family hydrolase | WD0802 | non-coding | Partial | 359 |
| amidophosphoribosyltransferase | WD1109 | Non-coding | Full | 1389 |
| aminotransferase, class V | WD0705 | coding | Full | 1130 |
| ankyrin repeat-containing prophage LambdaW1 | WD0636 | coding | Full | 750 |
| ankyrin repeat-containing prophage LambdaW1 | WD0637 | Non-coding | Full | 466 |
| ankyrin repeat-containing protein | WD0766 | coding | Full | 1165 |
| ankyrin repeat-containing protein | WD0073 | coding | Full | 770 |
| ankyrin repeat-containing protein | WD0073 | Non-coding | Full | 259 |
| ankyrin repeat-containing protein | WD0766 | coding | Full | 249 |
| ankyrin repeat-containing protein | WD0073 | coding | Full | 129 |
| ankyrin repeat-containing protein | WD0073 | non-coding | Partial | 259 |
| ankyrin repeat-containing protein | WD0441 | non-coding | Partial | 300 |
| ankyrin repeat-containing protein | WD0035 | non-coding | Partial | 499 |
| ApaG | WD1141 | coding | Full | 258 |
| araM protein | WD0787 | Non-coding | Full | 1010 |
| aspartyl/glutamyl-tRNA amidotransferase subunit B | WD0146 | coding | Full | 1423 |
| aspartyl-tRNA synthetase | WD0413 | Non-coding | Full | 1802 |
| ATP synthase F1, delta subunit | WD0656 | Non-coding | Full | 562 |
| ATP synthase F1, gamma subunit | WRi_012070 | non-coding | Partial | 560 |
| ATP-dependent Clp protease, ATP-binding subunit ClpA | WRi_012110 | coding | Full | 2284 |
| ATP-dependent Clp protease, proteolytic subunit ClpP | WD0319 | Non-coding | Full | 627 |
| ATP-dependent DNA helicase RecG | WD0824 | coding | Full | 2022 |
| ATP-dependent protease ATP-binding subunit ClpX | WD0318 | Non-coding | Full | 1275 |
| ATP-dependent protease ATP-binding subunit HslU | WD1190 | non-coding | Partial | 1152 |
| ATP-dependent protease La | WD0317 | non-coding | Partial | 2260 |
| ATP-dependent RNA helicase, DeaD/DeaH box family | WRi_012100 | coding | Full | 1197 |
| bifunctional proline dehydrogenase/pyrroline-5-carboxylate dehydrogenase | WD0103 | coding | Full | 1564 |
| carbamoyl phosphate synthase small subunit | WD0684 | Non-coding | Partial | 472 |
| carbamoyl phosphate synthase small subunit | WD0684 | Non-coding | Partial | 462 |
| cation ABC transporter, permease protein, putative | WD0362 | coding | Full | 498 |
| CBS domain-containing protein | WD0558 | Non-coding | Full | 1265 |
| CDP-diacylglycerol--serine O-phosphatidyltransferase, putative | WRi_010810 | non-coding | Partial | 189 |
| cell cycle transcriptional regulator | WD0732 | coding | Full | 771 |
| cell division protein FtsK, putative | WD0120 | Non-coding | Full | 594 |
| cell division protein FtsK, putative | WD0120 | Non-coding | Full | 538 |
| cell division protein FtsK, putative | WD0120 | non-coding | Partial | 942 |
| cell division protein FtsQ, putative | WD0096 | coding | Full | 773 |
| cell division protein FtsQ, putative | WD0096 | Non-coding | Partial | 672 |
| cell division protein FtsW, putative | WD0394 | non-coding | Partial | 989 |
| chromosomal DNA replication initiator-related protein | WRi_009920 | Non-coding | Full | 636 |
| Citrate synthase | WRi_009870 | Non-coding | Full | 1256 |
| conserved hypothetical protein | WRi_010780 | coding | Full | 560 |
| conserved hypothetical protein | WRi_010980 | coding | Full | 333 |
| Coq7 family protein | WD1100 | coding | Full | 543 |
| crossover junction endodeoxyribonuclease RuvC | WD0142 | Non-coding | Full | 289 |
| CTP synthetase | WD0468 | coding | Full | 1605 |
| cysteine desulfurase | WD0997 | coding | Full | 1146 |
| cytidine and deoxycytidylate deaminase family protein | WD0469 | Non-coding | Partial | 355 |
| cytochrome c biogenesis protein CcmA | WD0411 | coding | Full | 696 |
| cytochrome c family protein | WD0803 | Non-coding | Full | 528 |
| cytochrome c oxidase, subunit III | WD0141 | coding | Full | 816 |
| cytochrome c-type biogenesis protein CcmH, putative | WD0844 | coding | Full | 378 |
| cytochrome d ubiquinol oxidase, subunit I | WD0740 | Non-coding | Full | 1294 |
| cytochrome d ubiquinol oxidase, subunit II | WD0741 | Non-coding | Partial | 995 |
| D-alanine--D-alanine ligase | WD0095 | Non-coding | Full | 1041 |
| D-alanyl-D-alanine carboxypeptidase | WD0098 | coding | Full | 915 |
| deoxyguanosinetriphosphate triphosphohydrolase, putative | WD0709 | coding | Full | 1200 |
| Diacylglycerol kinase | WRi_011390 | non-coding | Partial | 310 |
| dihydrodipicolinate synthase | WD0775 | coding | Full | 882 |
| dihydroorotate dehydrogenase 2 | WD1239 | coding | Full | 957 |
| dihydropteroate synthase, putative | WD0883 | coding | Full | 399 |
| dimethyladenosine transferase | WD0090 | coding | Full | 583 |
| DNA gyrase subunit A | WD1202 | non-coding | Partial | 1390 |
| DNA gyrase, B subunit | WD0112 | Non-coding | Partial | 1507 |
| DNA ligase, NAD-dependent | WD0776 | Non-coding | Full | 1860 |
| DNA polymerase I | WD0658 | non-coding | Partial | 2074 |
| DNA polymerase III, alpha subunit | WD0780 | Non-coding | Full | 3235 |
| DNA polymerase III, beta subunit | WD1067 | coding | Full | 1158 |
| DNA polymerase III, epsilon subunit | WD0108 | Non-coding | Full | 634 |
| DNA polymerase III, gamma/tau subunit | WRi_010970 | Non-coding | Full | 1603 |
| DNA polymerase III, subunit, putative | WD0819 | Non-coding | Full | 776 |
| DNA processing chain A | WD0092 | Non-coding | Full | 1082 |
| DNA repair protein RadC | WD0357 | coding | Full | 678 |
| DNA-directed RNA polymerase subunit alpha | WD0658 | Non-coding | Full | 1068 |
| DNA-directed RNA polymerase, beta/beta' subunits | WD0024 | Non-coding | Full | 8481 |
| DnaJ domain protein | WRi_010880 | coding | Full | 1044 |
| dnaK suppressor protein, putative | WD1094 | coding | Full | 375 |
| dnaN | WD1067 | coding | Full | 307 |
| DsbB family disulfide bond formation protein | WD1099 | coding | Full | 522 |
| elongation factor G | WD0016 | coding | Full | 2073 |
| elongation factor Ts | WD0531 | coding | Full | 852 |
| elongation factor Tu | WD0017 | coding | Full | 584 |
| elongation factor Tu | WD0017 | coding | Full | 542 |
| elongation factor Tu | WD0683 | non-coding | Partial | 653 |
| elongation factor Tu | WD0683 | non-coding | Partial | 458 |
| endo/excinuclease amino terminal domain-containing protein | WD0358 | coding | Full | 291 |
| endo/excinuclease amino terminal domain-containing protein | WD0358 | non-coding | Partial | 256 |
| endonuclease III | WD0789 | non-coding | Partial | 647 |
| endopeptidase-related protein | WD0210 | coding | Full | 585 |
| excinuclease ABC subunit B | WD0839 | Non-coding | Full | 1914 |
| exodeoxyribonuclease III | WD1001 | non-coding | Partial | 778 |
| F0F1 ATP synthase subunit alpha | WD0655 | coding | Full | 1530 |
| F0F1 ATP synthase subunit beta | WD0203 | non-coding | Partial | 289 |
| FAD-dependent thymidylate synthase | WD1198 | non-coding | Partial | 723 |
| ferredoxin, 4Fe-4S | WD0093 | Non-coding | Full | 261 |
| ferredoxin, iron-sulfur cluster assembly system | WD0846 | coding | Full | 360 |
| Fic family protein | WD0365 | non-coding | Partial | 266 |
| folylpolyglutamate synthase | WRi_010890 | coding | Full | 1291 |
| fructose-bisphosphate aldolase | WD1238 | coding | Full | 841 |
| geranyltranstransferase | WD1192 | coding | Full | 1010 |
| glucosamine--fructose-6-phosphate aminotransferase (isomerizing) | WD0535 | coding | Full | 1812 |
| glutamyl-tRNA synthetase | WD077 | Non-coding | Full | 1250 |
| glutamyl-tRNA(Gln) amidotransferase, C subunit, putative | WD0037 | coding | Full | 354 |
| glutaredoxin family protein | WD0758 | coding | Full | 339 |
| glyceraldehyde 3-phosphate dehydrogenase | WD0451 | non-coding | Partial | 805 |
| glycerol-3-phosphate dehydrogenase (NAD+) | WD0731 | coding | Full | 983 |
| glycyl-tRNA synthetase, beta subunit | WD0155 | non-coding | Partial | 695 |
| GMP synthase | WD0195 | coding | Full | 1586 |
| GTP cyclohydrolase II | WRi_000040 | coding | Full | 1080 |
| GTP-binding protein EngA | WD1098 | coding | Full | 1321 |
| guanylate kinase | WD0439 | coding | Full | 639 |
| heat shock protein 90 | WD1277 | Non-coding | Full | 1001 |
| heat shock protein 90 | WD1277 | Non-coding | Partial | 609 |
| heat shock protein GrpE | WD0800 | coding | Full | 570 |
| heat shock sigma factor RpoH | WD1064 | coding | Full | 900 |
| helicase II - UvrD/PcrA | WD0963 | non-coding | Partial | 969 |
| heme exporter protein CcmB | WD1093 | coding | Full | 570 |
| heme exporter protein CcmC | WD0340 | Non-coding | Full | 634 |
| HesB/YadR/YfhF family protein | WD0708 | coding | Full | 462 |
| hexapeptide transferase family protein | WD0466 | Non-coding | Full | 390 |
| hflC protein | WD0832 | coding | Full | 873 |
| hflK protein | WD0831 | Non-coding | Full | 1034 |
| histidyl-tRNA synthetase | WD1076 | coding | Full | 1192 |
| HK97 family phage major capsid protein | WD0458 | non-coding | Partial | 1105 |
| HlyD family secretion protein | WD0649 | Non-coding | Full | 1592 |
| Holliday junction DNA helicase RuvA | WD1113 | Non-coding | Full | 527 |
| Holliday junction DNA helicase RuvB | WD1112 | Non-coding | Full | 989 |
| holo-(acyl-carrier-protein) synthase | WD0814 | coding | Full | 372 |
| HU family DNA-binding protein | WD1089 | coding | Full | 279 |
| hypothetical protein | WD1082 | coding | Full | 2567 |
| hypothetical protein | WD0745 | coding | Full | 1944 |
| hypothetical protein | WD0315 | coding | Full | 1224 |
| hypothetical protein | WRi_009930 | coding | Full | 1057 |
| hypothetical protein | WD0845 | coding | Full | 1041 |
| hypothetical protein | WD0364 | coding | Full | 993 |
| hypothetical protein | WD0249 | coding | Full | 927 |
| hypothetical protein | WD1191 | coding | Full | 877 |
| hypothetical protein | WD0757 | coding | Full | 871 |
| hypothetical protein | WD0773 | coding | Full | 828 |
| hypothetical protein | WD0094 | coding | Full | 820 |
| hypothetical protein | WD0335 | coding | Full | 806 |
| hypothetical protein | WD1137 | coding | Full | 801 |
| hypothetical protein | WD0975 | coding | Full | 800 |
| hypothetical protein | WD1104 | coding | Full | 791 |
| hypothetical protein | WD0989 | coding | Full | 782 |
| hypothetical protein | WD0632 | coding | Full | 781 |
| hypothetical protein | WD1002 | coding | Full | 750 |
| hypothetical protein | WD0702 | coding | Full | 738 |
| hypothetical protein | WD0284 | coding | Full | 728 |
| hypothetical protein | WD0706 | coding | Full | 699 |
| hypothetical protein | WD0811 | coding | Full | 648 |
| hypothetical protein | WD0771 | coding | Full | 631 |
| hypothetical protein | WD0632 | coding | Full | 627 |
| hypothetical protein | WRi_010900 | coding | Full | 591 |
| hypothetical protein | WD0341 | coding | Full | 590 |
| hypothetical protein | WD0284 | coding | Full | 566 |
| hypothetical protein | WD1242 | coding | Full | 557 |
| hypothetical protein | WD1242 | coding | Full | 537 |
| hypothetical protein | WRi_001340 | coding | Full | 537 |
| hypothetical protein | WD0049 | coding | Full | 531 |
| hypothetical protein | WD0117 | coding | Full | 529 |
| hypothetical protein | WD1065 | coding | Full | 510 |
| hypothetical protein | WD0034 | coding | Full | 502 |
| hypothetical protein | WD0999 | coding | Full | 498 |
| hypothetical protein | WD0643 | coding | Full | 473 |
| hypothetical protein | WD0991 | coding | Full | 470 |
| hypothetical protein | WD0143 | coding | Full | 468 |
| hypothetical protein | WD0827 | coding | Full | 459 |
| hypothetical protein | WD0069 | coding | Full | 457 |
| hypothetical protein | WD0863 | coding | Full | 456 |
| hypothetical protein | WD0635 | coding | Full | 441 |
| hypothetical protein | WD0412 | coding | Full | 432 |
| hypothetical protein | WD0128 | coding | Full | 427 |
| hypothetical protein | WD0764 | coding | Full | 417 |
| hypothetical protein | WD0771 | coding | Full | 402 |
| hypothetical protein | WD0716 | coding | Full | 393 |
| hypothetical protein | WRi_000670 | coding | Full | 390 |
| hypothetical protein | WD0332 | coding | Full | 383 |
| hypothetical protein | WD0284 | Non-coding | Partial | 370 |
| hypothetical protein | WD1229 | coding | Full | 369 |
| hypothetical protein | WD0361 | coding | Full | 366 |
| hypothetical protein | WD0033 | coding | Full | 348 |
| hypothetical protein | WD1088 | coding | Full | 327 |
| hypothetical protein | WD0715 | coding | Full | 285 |
| hypothetical protein | WD0557 | coding | Full | 267 |
| hypothetical protein | WRi_001880 | coding | Full | 264 |
| hypothetical protein | WD1082 | coding | Full | 258 |
| hypothetical protein | WD0034 | coding | Full | 194 |
| hypothetical protein | WRi_001890 | coding | Full | 138 |
| hypothetical protein | WRi_007740 | coding | Full | 118 |
| hypothetical protein | WD0061 | Non-coding | Partial | 117 |
| hypothetical protein | WD1278 | non-coding | Partial | 956 |
| hypothetical protein | WD0211 | non-coding | Partial | 834 |
| hypothetical protein | WD0854 | non-coding | Partial | 811 |
| hypothetical protein | WRi_012020 | non-coding | Partial | 801 |
| hypothetical protein | WD0686 | non-coding | Partial | 716 |
| hypothetical protein | WRi_000680 | non-coding | Partial | 711 |
| hypothetical protein | WD0996 | non-coding | Partial | 690 |
| hypothetical protein | WD0332 | non-coding | Partial | 575 |
| hypothetical protein | WD0823 | non-coding | Partial | 568 |
| hypothetical protein | WD1242 | coding | Full | 537 |
| hypothetical protein | WD0209 | non-coding | Partial | 511 |
| hypothetical protein | WD0284 | non-coding | Partial | 502 |
| hypothetical protein | WD0382 | non-coding | Partial | 483 |
| hypothetical protein | WD0632 | non-coding | Partial | 432 |
| hypothetical protein | WRi_007730 | non-coding | Partial | 432 |
| hypothetical protein | WD0523 | non-coding | Partial | 405 |
| hypothetical protein | WD0079 | non-coding | Partial | 374 |
| hypothetical protein | WD0284 | non-coding | Partial | 370 |
| hypothetical protein | WD0696 | non-coding | Partial | 337 |
| hypothetical protein | WRi_012020 | non-coding | Partial | 331 |
| hypothetical protein | WD0733 | non-coding | Partial | 314 |
| hypothetical protein | WD0332 | non-coding | Partial | 295 |
| hypothetical protein | WD0395 | non-coding | Partial | 244 |
| hypothetical protein | WRi_001500 | non-coding | Partial | 242 |
| hypothetical protein | WD1232 | non-coding | Partial | 241 |
| hypothetical protein | WD0835 | non-coding | Partial | 210 |
| hypothetical protein | WD0964 | non-coding | Partial | 204 |
| hypothetical protein | WD1229 | non-coding | Partial | 193 |
| hypothetical protein | WRi_010960 | non-coding | Partial | 173 |
| hypothetical protein | WD0823 | non-coding | Partial | 154 |
| hypothetical protein | WRi_004940 | coding | Full | 351 |
| insulinase family protease | WD0761 | Non-coding | Full | 1152 |
| iron compound ABC transporter, periplasmic iron compound-binding protein | WD0897 | coding | Full | 163 |
| iron compound ABC transporter, permease protein, putative | WD1136 | Non-coding | Full | 1628 |
| IS4 family transposase | WD0563 | Non-coding | Full | 672 |
| IS4 family transposase | WD0563 | non-coding | Partial | 588 |
| IS5 family transposase | WD0947 | non-coding | Partial | 539 |
| IS5 family transposase | WD0947 | non-coding | Partial | 272 |
| IS5 family transposase OrfB | WD1226 | Non-coding | Full | 242 |
| IS5 family transposase OrfB | WD1226 | Non-coding | Full | 199 |
| isocitrate dehydrogenase | WD0791 | Non-coding | Full | 1067 |
| isoleucyl-tRNA synthetase | WD0423 | non-coding | Partial | 824 |
| isoleucyl-tRNA synthetase | WD0423 | non-coding | Partial | 355 |
| leucyl-tRNA synthetase | WD0423 | non-coding | Partial | 1880 |
| lipoprotein signal peptidase | WD0760 | coding | Full | 477 |
| lipoyl synthase | WD0392 | coding | Full | 864 |
| lysyl-tRNA synthetase | WD0860 | coding | Full | 1536 |
| M16 family peptidase putative | WD0762 | Non-coding | Full | 1332 |
| M16 family peptidase putative | WD0737 | coding | Full | 717 |
| M16 family peptidase putative | WD0737 | Non-coding | Full | 397 |
| M48 family peptidase | WD0652 | Non-coding | Full | 1258 |
| maf protein | WD0333 | coding | Full | 597 |
| magnesium transporter | WD0375 | non-coding | Partial | 669 |
| major facilitator family transporter | WD0414 | coding | Full | 1260 |
| major facilitator family transporter | WD0470 | non-coding | Partial | 1247 |
| mannose-1-phosphate guanylyltransferase, interruption-C | WD1227 | non-coding | Partial | 735 |
| membrane-associated zinc metalloprotease, putative | WD1086 | coding | Full | 1105 |
| Metallophosphoesterase | WRi_000020 | coding | Full | 1101 |
| methionyl-tRNA formyltransferase | WD0866 | Non-coding | Full | 884 |
| methylenetetrahydrofolate dehydrogenase/methenyltetrahydrofolate cyclohydrolase | WD0555 | non-coding | Partial | 808 |
| methyltransferase, putative | WD1091 | coding | Full | 457 |
| methyltransferase, putative | WD0852 | non-coding | Partial | 508 |
| modification methylase, HemK family | WD0010 | coding | Full | 852 |
| Multisubunit Na+/H+ antiporter, MnhD subunit | WRi_007820 | non-coding | Partial | 480 |
| Na+/H+ antiporter family protein | WD0316 | Non-coding | Full | 670 |
| NADH dehydrogenase subunit D | WD0560 | non-coding | Partial | 514 |
| NADH dehydrogenase subunit E | WD0734 | coding | Full | 501 |
| NADH dehydrogenase subunit G | WD0160 | non-coding | Partial | 875 |
| NifR3 family protein | WD0025 | Non-coding | Full | 586 |
| NifU domain-containing protein | WD1075 | coding | Full | 585 |
| nuclease-related protein | WD1243 | non-coding | Partial | 449 |
| octaprenyl-diphosphate synthase | WD0799 | coding | Full | 984 |
| Type IV secretion system protein VirB10 | WD0006 | coding | Full | 954 |
| orotidine 5`-phosphate decarboxylase | WD0461 | non-coding | Partial | 618 |
| outer membrane protein TolC, putative | WD0068 | coding | Full | 1161 |
| outer membrane protein TolC, putative | WD0068 | non-coding | Partial | 1101 |
| pentapeptide repeat-containing protein | WD0440 | non-coding | Partial | 897 |
| pentapeptide repeat-containing protein | WD0440 | non-coding | Partial | 762 |
| peptidase, M22 family protein | WD0699 | coding | Full | 1008 |
| peptidyl-prolyl cis-trans isomerse D, putative | WD0797 | coding | Full | 1809 |
| periplasmic divalent cation tolerance protein | WD0828 | Non-coding | Full | 346 |
| phage uncharacterized protein | WD1016 | Non-coding | Partial | 565 |
| phenylacrylic acid decarboxylase, 3-octaprenyl-4-hydroxybenzoate carboxy-lyase | WD0556 | coding | Full | 576 |
| phosphate ABC transporter, permease protein, putative | WD0202 | Non-coding | Full | 739 |
| phosphatidate cytidylyltransferase | WD0526 | non-coding | Partial | 491 |
| phosphatidylglycerophosphatase A, putative | WD0730 | coding | Full | 483 |
| phosphatidylserine decarboxylase | WRi_010820 | Non-coding | Full | 700 |
| phosphoglucomutase/phosphomannomutase family protein | WD0695 | non-coding | Partial | 749 |
| phospho-N-acetylmuramoyl-pentapeptide- transferase | WD1102 | Non-coding | Full | 1030 |
| phosphoribosylamine--glycine ligase | WD0029 | coding | Full | 1272 |
| phosphoribosylaminoimidazole carboxylase, ATPasesubunit | WD1142 | coding | Full | 1065 |
| phosphoribosylaminoimidazolecarboxamide formyltransferase/IMP cyclohydrolase | WD0867 | coding | Full | 1512 |
| phosphoribosylglycinamide formyltransferase, putative | WD0763 | coding | Full | 561 |
| pmbA protein | WRi_012080 | non-coding | Partial | 985 |
| porphobilinogen deaminase | WD0542 | non-coding | Partial | 719 |
| preprotein translocase subunit SecB | WD0106 | Non-coding | Full | 378 |
| preprotein translocase subunit SecG | WD0467 | Non-coding | Full | 365 |
| preprotein translocase, SecA subunit | WRi_003630 | non-coding | Partial | 429 |
| prolipoprotein diacylglyceryl transferase | WD0768 | coding | Full | 792 |
| prophage LambdaW1, baseplate assembly protein J,putative | WD0283 | coding | Full | 533 |
| prophage LambdaW1, site-specific recombinase resolvase family protein | WD0288 | non-coding | Partial | 594 |
| prophage LambdaW1, site-specific recombinase resolvase family protein | WD0288 | non-coding | Partial | 255 |
| prophage LambdaW5, baseplate assembly protein J,putative | WD0639 | non-coding | Partial | 372 |
| prophage LambdaW5, baseplate assembly protein V | WD0642 | coding | Full | 716 |
| prophage LambdaW5, baseplate assembly protein W,putative | WD0640 | coding | Full | 336 |
| prophage LambdaW5, baseplate assembly protein W,putative | WD0640 | coding | Full | 234 |
| protease DO | WD0833 | non-coding | Partial | 761 |
| protease DO | WD0833 | non-coding | Partial | 740 |
| putative amino acid/peptide transporter | WD0422 | coding | Full | 1473 |
| putative monovalent cation/H+ antiporter subunitD | WD1081 | Non-coding | Full | 1560 |
| putative monovalent cation/H+ antiporter subunitD | WD1107 | non-coding | Partial | 478 |
| putative monovalent cation/H+ antiporter subunitD | WD1107 | non-coding | Partial | 351 |
| putative monovalent cation/H+ antiporter subunitE | WD0765 | coding | Full | 375 |
| pyruvate dehydrogenase subunit beta | WD0473 | coding | Full | 992 |
| pyruvate phosphate dikinase | WD0690 | coding | Full | 2808 |
| queuine tRNA-ribosyltransferase | WD0735 | Non-coding | Full | 1049 |
| recombinase A | WRi_010830 | non-coding | Partial | 338 |
| replicative DNA helicase | WD0354 | non-coding | Partial | 975 |
| response regulator PleD | WD0221 | non-coding | Partial | 539 |
| riboflavin biosynthesis protein RibD | WD0710 | coding | Full | 1063 |
| riboflavin biosynthesis protein RibF | WD0759 | coding | Full | 877 |
| ribonuclease HII | WD1103 | coding | Full | 597 |
| ribonuclease III | WD1240 | Non-coding | Full | 706 |
| ribonuclease III | WD1240 | non-coding | Partial | 288 |
| ribonucleotide-diphosphate reductase subunit alpha | WD0197 | non-coding | Partial | 1182 |
| ribonucleotide-diphosphate reductase subunit beta | WD0212 | coding | Full | 990 |
| ribose-phosphate pyrophosphokinase | WD0036 | coding | Full | 927 |
| ribosomal binding factor A | WD1317 | Non-coding | Full | 399 |
| ribosomal large subunit pseudouridine synthase C, putative | WD0415 | coding | Full | 357 |
| ribosomal large subunit pseudouridine synthase, RluD subfamily | WRi_011380 | non-coding | Partial | 629 |
| ribosomal protein L34 | WRi_001900 | coding | Full | 135 |
| ribosomal protein L35 | WD0864 | coding | Full | 207 |
| ribosomal protein S20 | WRi_009880 | Non-coding | Partial | 265 |
| ribosomal RNA large subunit methyltransferase J, putative | WD0070 | non-coding | Full | 580 |
| ribulose-phosphate 3-epimerase | WD0712 | coding | Full | 678 |
| rod shape-determining protein RodA | WD1108 | coding | Full | 1052 |
| S-adenosylmethionine synthetase | WD0136 | non-coding | Partial | 291 |
| SCO1/SenC family protein | WD0109 | coding | Full | 582 |
| seryl-tRNA synthetase | WD0028 | coding | Full | 1299 |
| signal recognition particle protein | WD1080 | Non-coding | Partial | 1190 |
| single-strand binding protein | WD0774 | coding | Full | 483 |
| single-stranded-DNA-specific exonuclease RecJ | WD0312 | Non-coding | Full | 1740 |
| site-specific recombinase, phage integrase family | WRi_009900 | Non-coding | Full | 960 |
| small multidrug resistance protein | WRi_001520 | coding | Full | 312 |
| Small ribosomal subunit (16S rRNA) | Wp16SA | rRNA | rRNA | 39 |
| SNF2 family helicase | WD0610 | non-coding | Partial | 744 |
| SNF2 family helicase | WD0610 | non-coding | Partial | 179 |
| SNF2 family helicase | WD0610 | non-coding | Partial | 210 |
| sodium/alanine symporter family protein | WRi_010790 | non-coding | Partial | 1073 |
| sodium/alanine symporter family protein, putative | WD0330 | Non-coding | Full | 1338 |
| SsrA-binding protein | WD0767 | coding | Full | 450 |
| stationary-phase survival protein SurE | WD1077 | coding | Full | 753 |
| Sua5/YciO/YrdC/YwlC family protein | WD0820 | Non-coding | Full | 589 |
| succinate dehydrogenase iron-sulfur subunit | WD0727 | non-coding | Partial | 399 |
| succinate dehydrogenase, cytochrome b556 subunit | WRi_012000 | Non-coding | Full | 377 |
| succinate dehydrogenase, hydrophobic membrane anchor protein | WRi_012010 | Non-coding | Full | 347 |
| succinyl-diaminopimelate desuccinylase | WD0788 | coding | Full | 1188 |
| sugE protein | WRi_001510 | Non-coding | Full | 345 |
| superoxide dismutase, Fe | WD0738 | Non-coding | Full | 376 |
| surface antigen | WD1085 | Non-coding | Full | 1337 |
| surface antigen | WD1085 | coding | Full | 1026 |
| Surface antigen Wsp | WD1063 | Non-coding | Partial | 56 |
| TenA family transcription regulator | WD0139 | coding | Full | 684 |
| TenA family transcription regulator | WD0139 | coding | Full | 588 |
| TerC family membrane protein | WD0194 | non-coding | Partial | 429 |
| tldD protein | WD0998 | Non-coding | Full | 1412 |
| TPR domain protein Partial | WD0003 | Non-coding | Full | 1076 |
| TRAM domain-containing protein | WD0421 | Non-coding | Full | 770 |
| transcription antitermination protein NusG, putative | WD0421 | coding | Full | 832 |
| transcription elongation factor GreA | WD0654 | Non-coding | Full | 494 |
| transcription elongation factor NusA | WD1319 | Non-coding | Full | 1560 |
| transcription termination factor Rho | WD0795 | coding | Full | 1404 |
| transketolase | WD0387 | Non-coding | Full | 2035 |
| translation initiation factor IF-1 | WD0334 | coding | Full | 264 |
| translation initiation factor IF-2 | WD1318 | non-coding | Partial | 779 |
| translation initiation factor IF-2 | WD1318 | non-coding | Partial | 434 |
| translation initiation factor IF-3 | WD0977 | coding | Full | 517 |
| translocation protein TolB | WD0038 | Non-coding | Partial | 163 |
| transposase | WD0907 | Non-coding | Full | 258 |
| transposase | WRi_008070 | Non-coding | Full | 303 |
| transposase ISWPi3 | WRi_p04420 | Non-coding | Full | 310 |
| transposase, | WRi_p08080 | Non-coding | Full | 813 |
| transposase | WD0907 | Non-coding | Full | 381 |
| transposase, ISWpi3 | WRi_p04420 | non-coding | Partial | 401 |
| transposase, ISWpi9 | WRi_p04780 | Non-coding | Full | 533 |
| trigger factor, putative | WD0320 | non-coding | Partial | 413 |
| triosephosphate isomerase | WD0091 | coding | Full | 729 |
| tRNA delta(2)-isopentenylpyrophosphate transferase | WD0822 | coding | Full | 873 |
| tRNA pseudouridine synthase A | WD1068 | Non-coding | Full | 740 |
| tRNA-Ala | WRi_t09220 | tRNA | tRNA | 76 |
| tRNA-Arg | WRi_t00350 | tRNA | tRNA | 78 |
| tRNA-Arg | WRi_t06170 | tRNA | tRNA | 74 |
| tRNA-Gly | WRi_t05220 | tRNA | tRNA | 71 |
| tRNA-Leu | WRi_t04010 | tRNA | tRNA | 87 |
| tRNA-Leu | WRi_t09470 | tRNA | tRNA | 85 |
| tRNA-Leu | WRi_t00030 | tRNA | tRNA | 85 |
| tRNA-Leu | WRi_t04320 | tRNA | tRNA | 83 |
| tRNA-Lys | WRi_t09230 | tRNA | tRNA | 73 |
| tRNA-Met | WRi_t06270 | tRNA | tRNA | 74 |
| tRNA-Ser | WRi_t07290 | tRNA | tRNA | 87 |
| tRNA-Thr | WRi_t01600 | tRNA | tRNA | 71 |
| tRNA-Trp | WRi_t00160 | tRNA | tRNA | 73 |
| tRNA-Tyr | WRi_t05230 | tRNA | tRNA | 83 |
| tRNA-Val | WRi_t11810 | tRNA | tRNA | 73 |
| tryptophanyl-tRNA synthetase | WD0801 | coding | Full | 1000 |
| type I secretion system ATPase | WD0770 | Non-coding | Full | 1713 |
| type IV secretion system ATPase VirB4 | WD0858 | Non-coding | Full | 2412 |
| type IV secretion system protein VirB10 | WD0006 | non-coding | Full | 1432 |
| type IV secretion system protein VirB9 | WD0005 | Coding | Full | 801 |
| type IV secretion system protein VirB11 | WRi_000080 | Non-coding | Full | 992 |
| type IV secretion system protein VirB6 | WRi_008190 | Non-coding | Partial | 1402 |
| type IV secretion system protein VirB6 | WD0855 | Non-coding | Partial | 1832 |
| type IV secretion system protein VirB6 | WD0855 | Non-coding | Partial | 1121 |
| type IV secretion system protein VirB6 | WD0857 | Non-coding | Partial | 857 |
| type IV secretion system protein VirB8, putative | WRi_007830 | Non-coding | Partial | 387 |
| ubiquinol-cytochrome c reductase, cytochrome b | WD1071 | coding | Full | 1230 |
| ubiquinol-cytochrome c reductase, cytochrome c1 | WD1070 | coding | Full | 858 |
| ubiquinone/menaquinone biosynthesis methlytransferase UbiE | WD0393 | Non-coding | Full | 715 |
| UDP-N-acetylenolpyruvoylglucosamine reductase | WD0541 | Non-coding | Full | 837 |
| UDP-N-acetylglucosamine 1-carboxyvinyltransferase | WD1197 | Non-coding | Full | 784 |
| UDP-N-acetylglucosamine pyrophosphorylase | WD0133 | Non-coding | Full | 926 |
| UDP-N-acetylmuramoylalanine--D-glutamate ligase | WD0849 | Non-coding | Full | 1545 |
| undecaprenyl diphosphate synthase | WD0527 | non-coding | Partial | 617 |
| uridylate kinase | WD0530 | non-coding | Partial | 400 |
| uroporphyrinogen-III synthase, putative | WD1000 | coding | Full | 579 |
| UvrD/Rep/AddA family helicase | WD0359 | non-coding | Partial | 1952 |
| UvrD/Rep/AddA family helicase | WD0359 | non-coding | Partial | 956 |
| type IV secretion system protein VirD4 | WRi_000090 | non-coding | Partial | 1328 |
| YggT family protein | WRi_001280 | coding | Full | 279 |
