# Supplementary material for: Presence of Extensive Wolbachia Symbiont Insertions Discovered in the Genome of Its Host Glossina morsitans morsitans
Source: PLoS Negl Trop Dis. 2014 Apr 24;8(4):e2728. doi: 10.1371/journal.pntd.0002728 (PMC3998919; doi:10.1371/journal.pntd.0002728)
Supplement: Table S7 — Description of the second set of Wolbachia inserted regions into the G. m. morsitans chromosomes. (DOCX) [file pntd.0002728.s011.docx]

**Table S7.** Description of the second set of *Wolbachia* inserted regions (Insertion B) into the *G. m. morsitans* chromosomes.

| **Product** | **Homolog** | **Type** | **Insertion** | **Length** |
| --- | --- | --- | --- | --- |
| 1-acyl-sn-glycerol-3-phosphate acyltransferase family protein | WRi_009890 | Non-coding | Partial | 601 |
| adenylate kinase | WD0661 | non-coding | Partial | 478 |
| alanyl-tRNA synthetase | WRi_008260 | non-coding | Partial | 1286 |
| alanyl-tRNA synthetase | WRi_008260 | Non-coding | Partial | 659 |
| alanyl-tRNA synthetase | WD0862 | Non-coding | Partial | 250 |
| acetylornithine transaminase protein | WD0559 | non-coding | Partial | 1020 |
| carbamoyl phosphate synthase small subunit | WD0684 | coding | Full | 462 |
| malonyl-CoA decarboxylase, putative | WD0478 | non-coding | Partial | 1069 |
| cell division protein FtsW, putative | WD0394 | non-coding | Partial | 989 |
| hypothetical protein | WD0757 | non-coding | Partial | 861 |
| HK97 family phage major capsid protein | WD0458 | non-coding | Partial | 799 |
| TRAM domain-containing protein | WD0421 | non-coding | Partial | 770 |
| pentapeptide repeat-containing protein | WD0440 | non-coding | Partial | 612 |
| hypothetical protein | WD0382 | non-coding | Partial | 462 |
| hypothetical protein | WD0523 | coding | Partial | 405 |
| prophage LambdaW1, site-specific recombinase resolvase family protein | WD0288 | non-coding | Partial | 255 |
| prophage LambdaW1, site-specific recombinase resolvase family protein | WD0288 | non-coding | Partial | 568 |
| hypothetical protein | WD0395 | non-coding | Partial | 221 |
| phosphatidate cytidylyltransferase | WD0526 | non-coding | Partial | 626 |
| ATP-dependent Clp protease, ATP-binding subunit ClpA | WRi_012110 | non-coding | Partial | 1722 |
| ATP-dependent Clp protease, ATP-binding subunit ClpA | WRi_012110 | non-coding | Partial | 583 |
| periplasmic divalent cation tolerance protein | WD0828 | non-coding | Partial | 335 |
| DNA polymerase III, subunit, putative | WD0819 | non-coding | Partial | 689 |
| replicative DNA helicase | WD0354 | non-coding | Partial | 958 |
| DnaJ domain protein | WD1051 | coding | Partial | 120 |
| drug resistance transporter | WD0248 | non-coding | Partial | 850 |
| endo/excinuclease amino terminal domain-containing protein | WD0358 | non-coding | Partial | 256 |
| endopeptidase-related protein | WD0210 | non-coding | Partial | 583 |
| signal recognition particle protein | WD1080 | non-coding | Partial | 1192 |
| Fic family protein | WD0365 | coding | Partial | 215 |
| methylenetetrahydrofolate dehydrogenase/methenyltetrahydrofolate cyclohydrolase | WD0555 | non-coding | Partial | 749 |
| Folate synthesis bifunctional protein | WRi_008620 | non-coding | Partial | 763 |
| glyceraldehyde 3-phosphate dehydrogenase | WD0451 | non-coding | Partial | 805 |
| UDP-N-acetylglucosamine pyrophosphorylase | WD0133 | non-coding | Partial | 925 |
| glycyl-tRNA synthetase, beta subunit | WD0155 | non-coding | Partial | 695 |
| GMP synthase | WD0195 | non-coding | Partial | 1460 |
| DNA gyrase subunit A | WD1202 | non-coding | Partial | 978 |
| porphobilinogen deaminase | WD0542 | coding | Partial | 633 |
| ATP-dependent protease ATP-binding subunit HslU | WD1190 | non-coding | Partial | 1148 |
| heat shock protein 90 | WD1277 | non-coding | Partial | 610 |
| hypothetical protein | WD0855 | non-coding | Partial | 1835 |
| hypothetical protein | WD0999 | coding | Partial | 488 |
| hypothetical protein | WD0835 | non-coding | Partial | 210 |
| hypothetical protein | WD0733 | non-coding | Partial | 314 |
| hypothetical protein | WRi_009910 | non-coding | Partial | 121 |
| hypothetical protein | WD0632 | non-coding | Partial | 435 |
| hypothetical protein | WD0728 | non-coding | Partial | 941 |
| hypothetical protein | WD0854 | non-coding | Partial | 801 |
| hypothetical protein | WD0975 | non-coding | Partial | 791 |
| hypothetical protein | WD0818 | coding | Partial | 727 |
| hypothetical protein | WD0249 | non-coding | Partial | 709 |
| hypothetical protein | WD0996 | coding | Partial | 668 |
| hypothetical protein | WD1137 | non-coding | Partial | 584 |
| hypothetical protein | WD0332 | coding | Partial | 578 |
| hypothetical protein | WD0854 | coding | Partial | 555 |
| hypothetical protein | WD0284 | non-coding | Partial | 541 |
| hypothetical protein | WD1199 | coding | Partial | 463 |
| hypothetical protein | WD0855 | coding | Partial | 454 |
| hypothetical protein | WD0209 | non-coding | Partial | 407 |
| hypothetical protein | WD0079 | non-coding | Partial | 374 |
| hypothetical protein | WD0823 | non-coding | Partial | 126 |
| hypothetical protein | WRi_002030 | non-coding | Partial | 892 |
| hypothetical protein | WD0284 | coding | Partial | 501 |
| hypothetical protein | WD0284 | non-coding | Partial | 370 |
| hypothetical protein | WD0696 | non-coding | Partial | 337 |
| isoleucyl-tRNA synthetase | WD0423 | Non-coding | Partial | 826 |
| isoleucyl-tRNA synthetase | WD0423 | Non-coding | Partial | 429 |
| isoleucyl-tRNA synthetase | WD0423 | non-coding | Partial | 357 |
| translation initiation factor IF-2 | WD1318 | non-coding | Partial | 569 |
| translation initiation factor IF-2 | WD1318 | non-coding | Partial | 196 |
| iron compound ABC transporter, permease protein, putative | WD1136 | coding | Partial | 781 |
| IS4 family transposase | WD0563 | non-coding | Partial | 588 |
| IS4 family transposase | WD0563 | non-coding | Partial | 379 |
| IS5 family transposase | WD0947 | non-coding | Partial | 542 |
| IS5 family transposase | WD0947 | non-coding | Partial | 274 |
| 4-hydroxy-3-methylbut-2-en-1-yl diphosphate synthase | WD0116 | non-coding | Partial | 1294 |
| dimethyladenosine transferase | WRi_001610 | coding | Partial | 736 |
| leucyl-tRNA synthetase | WD0060 | non-coding | Partial | 818 |
| ATP-dependent protease La | WD0317 | non-coding | Partial | 2247 |
| major facilitator family transporter | WD0470 | non-coding | Partial | 1254 |
| mannose-1-phosphate guanylyltransferase, interruption-C | WD1227 | non-coding | Partial | 735 |
| Metallophosphoesterase | WRi_000020 | non-coding | Partial | 1265 |
| methyltransferase, putative | WD0852 | non-coding | Partial | 489 |
| S-adenosylmethionine synthetase | WD0136 | non-coding | Partial | 291 |
| magnesium transporter | WD0375 | non-coding | Partial | 669 |
| UDP-N-acetylglucosamine 1-carboxyvinyltransferase | WD1197 | non-coding | Partial | 1077 |
| ribonucleotide-diphosphate reductase subunit alpha | WD0197 | non-coding | Partial | 1035 |
| endonuclease III | WD0789 | non-coding | Partial | 644 |
| NADH dehydrogenase subunit D | WD0560 | non-coding | Partial | 525 |
| NADH dehydrogenase I, F subunit | WRi_009240 | non-coding | Partial | 380 |
| NADH dehydrogenase subunit G | WD0560 | non-coding | Partial | 875 |
| transcription elongation factor NusA | WRi_013490 | non-coding | Partial | 928 |
| phosphoglyceromutase | WRi_008330 | non-coding | Partial | 515 |
| phage uncharacterized protein | WD1016 | non-coding | Partial | 545 |
| phosphate ABC transporter, permease protein, putative | WRi_001910 | non-coding | Partial | 313 |
| phosphoglucomutase/phosphomannomutase family protein | WD0695 | non-coding | Partial | 752 |
| fatty acid/phospholipid synthesis protein | WRi_009340 | coding | Partial | 653 |
| DNA polymerase I | WD1003 | non-coding | Partial | 2063 |
| peptide chain release factor 1 | WD0247 | non-coding | Partial | 741 |
| prophage LambdaW1, site-specific recombinase resolvase family protein | WD0288 | non-coding | Partial | 568 |
| prophage LambdaW5, baseplate assembly protein J, putative | WD0639 | non-coding | Partial | 372 |
| prolyl-tRNA synthetase | WD0813 | Non-coding | Partial | 276 |
| adenylosuccinate synthetase | WD0337 | non-coding | Partial | 1272 |
| amidophosphoribosyltransferase | WD1109 | non-coding | Partial | 1025 |
| putative monovalent cation/H+ antiporter subunitD | WD1107 | coding | Partial | 346 |
| dihydroorotate dehydrogenase 2 | WD1239 | non-coding | Partial | 888 |
| orotidine 5`-phosphate decarboxylase | WD0461 | non-coding | Partial | 587 |
| rod shape-determining protein RodA | WD1108 | non-coding | Partial | 304 |
| DNA-directed RNA polymerase subunit alpha | WD0658 | non-coding | Partial | 390 |
| DNA-directed RNA polymerase subunit alpha | WD0658 | Non-coding | Partial | 280 |
| 30S ribosomal protein S4 | WD0388 | non-coding | Partial | 338 |
| 30S ribosomal protein S4 | WD0388 | non-coding | Partial | 153 |
| succinate dehydrogenase iron-sulfur subunit | WD0727 | non-coding | Partial | 394 |
| 2-oxoglutarate dehydrogenase, E2 component, dihydrolipoamide succinyltransferase | WD0544 | non-coding | Partial | 1106 |
| FAD-dependent thymidylate synthase | WD1198 | non-coding | Partial | 723 |
| TPR domain-containing protein | WD0198 | non-coding | Partial | 567 |
| transposase, degenerate; ISWpi3 | WRi_p03810 | non-coding | Partial | 1051 |
| trigger factor, putative | WD0320 | Non-coding | Partial | 411 |
| elongation factor Tu | WD0683 | non-coding | Partial | 653 |
| 4-hydroxybenzoate polyprenyltransferase | WD1316 | Non-coding | Partial | 695 |
| undecaprenyl diphosphate synthase | WD0527 | non-coding | Partial | 373 |
| uroporphyrinogen-III synthase, putative | WD1000 | non-coding | Partial | 539 |
| helicase II - UvrD/PcrA | WD0963 | non-coding | Partial | 482 |
| UvrD/Rep/AddA family helicase | WD0359 | non-coding | Partial | 1958 |
| UvrD/Rep/AddA family helicase | WD0359 | Non-coding | Partial | 947 |
| type IV secretion system protein VirB6 | WD0857 | non-coding | Partial | 468 |
| type IV secretion system protein VirB6 | WD0857 | Non-coding | Partial | 467 |
| type IV secretion system protein VirD4 | WRi_000090 | non-coding | Partial | 1328 |
| exodeoxyribonuclease III | WD1001 | non-coding | Partial | 779 |
| 3,4-dihydroxy-2-butanone 4-phosphate synthase | WD0653 | coding | Full | 645 |
| ferredoxin, 4Fe-4S | WD0093 | non-coding | Full | 257 |
| ABC transporter, periplasmic substrate-binding protein, putative | WD1105 | coding | Full | 456 |
| ABC transporter, ATP-binding protein | WD0990 | non-coding | Full | 708 |
| ABC transporter, ATP-binding protein | WD0707 | non-coding | Full | 478 |
| acetyltransferase | WD0729 | coding | Full | 807 |
| aconitate hydratase | WD0105 | non-coding | Full | 2158 |
| acyl carrier protein | WD1193 | coding | Full | 261 |
| holo-(acyl-carrier-protein) synthase | WD0814 | non-coding | Full | 372 |
| alpha/beta fold family hydrolase | WD0802 | non-coding | Full | 750 |
| aminotransferase, class V | WD0705 | contig | Full | 1130 |
| ankyrin repeat-containing protein | WD0766 | non-coding | Full | 1164 |
| ankyrin repeat-containing protein | WD0766 | Non-coding | Partial | 249 |
| ankyrin repeat-containing protein | WD0073 | non-coding | Full | 189 |
| ankyrin repeat-containing protein | WD0073 | non-coding | Partial | 117 |
| ankyrin repeat-containing protein | WD0073 | Non-coding | Full | 110 |
| ankyrin repeat-containing prophage LambdaW1 | WD0637 | coding | Full | 750 |
| ankyrin repeat-containing prophage LambdaW1 | WD0636 | non-coding | Full | 466 |
| ApaG | WD1141 | coding | Full | 258 |
| aspartyl-tRNA synthetase | WD0413 | non-coding | Full | 1780 |
| ATP-dependent RNA helicase, DeaD/DeaH box family | WRi_012100 | non-coding | Full | 1196 |
| F0F1 ATP synthase subunit alpha | WD0655 | coding | Full | 1530 |
| ATP synthase F1, delta subunit | WD0656 | non-coding | Full | 560 |
| carbamoyl phosphate synthase small subunit | WD0684 | coding | Full | 462 |
| cation ABC transporter, permease protein, putative | WD0362 | coding | Full | 498 |
| heme exporter protein CcmB | WD1093 | coding | Full | 570 |
| heme exporter protein CcmC | WD0340 | non-coding | Full | 634 |
| hypothetical protein | WD0631 | non-coding | Full | 1429 |
| CBS domain-containing protein | WD0558 | non-coding | Full | 1265 |
| major facilitator family transporter | WD0414 | non-coding | Full | 1262 |
| araM protein | WD0787 | non-coding | Full | 1012 |
| hypothetical protein | WD0041 | non-coding | Full | 944 |
| hypothetical protein | WD0632 | non-coding | Full | 781 |
| hypothetical protein | WD0686 | non-coding | Partial | 680 |
| IS4 family transposase | WD0563 | non-coding | Full | 673 |
| cytochrome c biogenesis protein CcmA | WD0411 | non-coding | Full | 624 |
| hypothetical protein | WD0412 | non-coding | Full | 432 |
| ribosomal large subunit pseudouridine synthase C, putative | WD0415 | coding | Full | 357 |
| hypothetical protein | WD0033 | coding | Full | 348 |
| hypothetical protein | WD0557 | coding | Full | 267 |
| IS5 family transposase OrfB | WD1226 | non-coding | Full | 396 |
| chromosomal DNA replication initiator-related protein | WRi_009920 | non-coding | Full | 634 |
| ATP-dependent Clp protease, proteolytic subunit ClpP | WD0319 | non-coding | Full | 627 |
| ATP-dependent protease ATP-binding subunit ClpX | WD0318 | non-coding | Full | 1273 |
| Coq7 family protein | WD1100 | coding | Full | 543 |
| cytochrome c oxidase, subunit III | WD0141 | coding | Full | 805 |
| cell cycle transcriptional regulator | WD0732 | coding | Full | 771 |
| cytochrome d ubiquinol oxidase, subunit I | WD0740 | non-coding | Full | 1294 |
| cytochrome d ubiquinol oxidase, subunit II | WD0741 | coding | Full | 835 |
| cytidine and deoxycytidylate deaminase family protein | WD0469 | coding | Full | 355 |
| cytochrome c family protein | WD0803 | non-coding | Full | 528 |
| cytochrome c-type biogenesis protein CcmH, putative | WD0844 | coding | Full | 378 |
| D-alanine--D-alanine ligase | WD0095 | non-coding | Full | 1021 |
| D-alanyl-D-alanine carboxypeptidase | WD0098 | non-coding | Full | 748 |
| dihydrodipicolinate synthase | WD0775 | non-coding | Full | 882 |
| 2,3,4,5-tetrahydropyridine-2,6-carboxylate N-succinyltransferase | WD0714 | non-coding | Full | 831 |
| succinyl-diaminopimelate desuccinylase | WD0788 | coding | Full | 1188 |
| deoxyguanosinetriphosphate triphosphohydrolase, putative | WD0709 | coding | Full | 1200 |
| DNA polymerase III, alpha subunit | WD0780 | non-coding | Full | 3236 |
| dnaK suppressor protein, putative | WD1094 | non-coding | Full | 326 |
| DNA polymerase III, beta subunit | WRi_011020 | coding | Full | 1158 |
| DNA polymerase III, epsilon subunit | WD0108 | non-coding | Full | 620 |
| DNA processing chain A | WD0092 | coding | Full | 1107 |
| DsbB family disulfide bond formation protein | WD1099 | non-coding | Full | 522 |
| 1-deoxy-D-xylulose 5-phosphate reductoisomerase | WD0992 | non-coding | Full | 1157 |
| endo/excinuclease amino terminal domain-containing protein | WD0358 | coding | Full | 291 |
| GTP-binding protein EngA | WD1098 | non-coding | Full | 1321 |
| 3-oxoacyl-(acyl-carrier-protein) synthase II | WD1194 | coding | Full | 1272 |
| 3-oxoacyl-(acyl-carrier-protein) reductase | WD0650 | non-coding | Full | 734 |
| (3R)-hydroxymyristoyl-ACP dehydratase | WD1083 | coding | Full | 432 |
| Na+/H+ antiporter family protein | WD0316 | non-coding | Full | 670 |
| ferredoxin, iron-sulfur cluster assembly system | WD0846 | coding | Full | 360 |
| methionyl-tRNA formyltransferase | WD0866 | non-coding | Full | 884 |
| fructose-bisphosphate aldolase | WD1238 | non-coding | Full | 841 |
| cell division protein FtsK, putative | WD0120 | non-coding | Full | 564 |
| cell division protein FtsK, putative | WD0120 | non-coding | Full | 472 |
| cell division protein FtsQ, putative | WD0096 | coding | Full | 444 |
| elongation factor G | WD0016 | coding | Full | 2091 |
| aspartyl/glutamyl-tRNA amidotransferase subunit B | WD0146 | non-coding | Full | 1424 |
| glucosamine--fructose-6-phosphate aminotransferase (isomerizing) | WD0535 | non-coding | Full | 1818 |
| Citrate synthase | WRi_009870 | non-coding | Full | 1250 |
| glutamyl-tRNA synthetase | WD0778 | non-coding | Full | 1252 |
| glutamyl-tRNA(Gln) amidotransferase, C subunit, putative | WRi_000310 | non-coding | Partial | 268 |
| glutaredoxin family protein | WD0758 | coding | Full | 339 |
| guanylate kinase | WD0439 | coding | Full | 636 |
| glycerol-3-phosphate dehydrogenase (NAD+) | WD0731 | coding | Full | 984 |
| transcription elongation factor GreA | WD0654 | non-coding | Full | 494 |
| heat shock protein GrpE | WD0800 | coding | Full | 570 |
| GTP cyclohydrolase II | WRi_000040 | non-coding | Full | 1073 |
| DNA gyrase, B subunit | WD0112 | non-coding | Full | 1503 |
| modification methylase, HemK family | WD0010 | coding | Full | 414 |
| HesB/YadR/YfhF family protein | WD0708 | coding | Full | 462 |
| hexapeptide transferase family protein | WD0466 | coding | Full | 390 |
| hflC protein | WD0832 | non-coding | Full | 875 |
| hflK protein | WD0706 | non-coding | Full | 1033 |
| HlyD family secretion protein | WD0649 | non-coding | Full | 1592 |
| heat shock protein 90 | WD1277 | coding | Full | 1002 |
| protease DO | WD0833 | non-coding | Full | 1455 |
| HU family DNA-binding protein | WD1089 | coding | Full | 279 |
| hypothetical protein | WD0856 | non-coding | Full | 2372 |
| hypothetical protein | WD1104 | non-coding | Full | 791 |
| hypothetical protein | WD0854 | non-coding | Full | 1197 |
| hypothetical protein | WD1082 | coding | Full | 258 |
| hypothetical protein | WD0856 | non-coding | Full | 292 |
| hypothetical protein | WD0764 | non-coding | Full | 265 |
| hypothetical protein | WD0635 | coding | Full | 441 |
| hypothetical protein | WD1082 | non-coding | Full | 2568 |
| hypothetical protein | WD0315 | coding | Full | 1224 |
| hypothetical protein | WD0845 | coding | Full | 1041 |
| hypothetical protein | WD0364 | coding | Full | 993 |
| hypothetical protein | WD1278 | non-coding | Partial | 947 |
| hypothetical protein | WD1191 | non-coding | Full | 872 |
| hypothetical protein | WD0773 | coding | Full | 828 |
| hypothetical protein | WD0335 | coding | Full | 807 |
| hypothetical protein | WD0094 | non-coding | Full | 806 |
| hypothetical protein | WD0989 | coding | Full | 783 |
| hypothetical protein | WD1002 | non-coding | Full | 749 |
| hypothetical protein | WD0811 | coding | Full | 648 |
| hypothetical protein | WD0771 | non-coding | Full | 631 |
| hypothetical protein | WD0796 | non-coding | Full | 609 |
| hypothetical protein | WD0341 | non-coding | Full | 590 |
| hypothetical protein | WD0117 | non-coding | Full | 532 |
| hypothetical protein | WD1030 | non-coding | Full | 485 |
| hypothetical protein | WD0991 | coding | Full | 470 |
| hypothetical protein | WD0143 | coding | Full | 468 |
| hypothetical protein | WD0827 | coding | Full | 459 |
| hypothetical protein | WD0069 | non-coding | Full | 457 |
| hypothetical protein | WD0863 | coding | Full | 456 |
| hypothetical protein | WD0854 | non-coding | Full | 428 |
| hypothetical protein | WD0128 | non-coding | Full | 427 |
| hypothetical protein | WD0332 | non-coding | Full | 410 |
| hypothetical protein | WD0853 | non-coding | Full | 402 |
| hypothetical protein | WD0771 | non-coding | Full | 402 |
| hypothetical protein | WD0807 | non-coding | Full | 397 |
| hypothetical protein | WD0332 | coding | Full | 383 |
| hypothetical protein | WD1084 | non-coding | Full | 370 |
| hypothetical protein | WD1229 | non-coding | Full | 369 |
| hypothetical protein | WD0361 | coding | Full | 366 |
| hypothetical protein | WD0853 | coding | Full | 338 |
| hypothetical protein | WD0854 | coding | Full | 334 |
| hypothetical protein | WD1088 | coding | Full | 327 |
| hypothetical protein | WD0835 | coding | Full | 313 |
| hypothetical protein | WRi_001880 | coding | Full | 264 |
| hypothetical protein | WD0034 | coding | Full | 194 |
| hypothetical protein | WD0745 | non-coding | Full | 1946 |
| hypothetical protein | WRi_009930 | non-coding | Full | 1052 |
| hypothetical protein | WD0702 | coding | Full | 738 |
| hypothetical protein | WD0706 | coding | Full | 699 |
| hypothetical protein | WD0748 | non-coding | Full | 659 |
| hypothetical protein | WD0696 | coding | Full | 652 |
| hypothetical protein | WD0717 | non-coding | Full | 618 |
| hypothetical protein | WRi_012170 | coding | Full | 537 |
| hypothetical protein | WD0049 | coding | Full | 531 |
| hypothetical protein | WRi_011010 | coding | Full | 498 |
| hypothetical protein | WD0643 | non-coding | Full | 475 |
| hypothetical protein | WD0412 | coding | Full | 432 |
| hypothetical protein | WD0764 | coding | Full | 417 |
| hypothetical protein | WD0716 | coding | Full | 393 |
| hypothetical protein | WRi_002110 | non-coding | Full | 296 |
| hypothetical protein | WD0715 | coding | Full | 285 |
| hypothetical protein |  | non-coding | Full | 172 |
| hypothetical protein | WRi_001890 | coding | Full | 138 |
| isoleucyl-tRNA synthetase | WD0423 | non-coding | Full | 885 |
| translation initiation factor IF-1 | WD0334 | coding | Full | 264 |
| insulinase family protease | WD0761 | non-coding | Full | 1144 |
| cysteine desulfurase | WD0997 | coding | Full | 1140 |
| isocitrate dehydrogenase | WD0791 | non-coding | Full | 1067 |
| geranyltranstransferase | WD1192 | coding | Full | 1008 |
| octaprenyl-diphosphate synthase | WD0799 | coding | Full | 984 |
| 4-diphosphocytidyl-2C-methyl-D-erythritol kinase | WD0360 | coding | Full | 645 |
| prolipoprotein diacylglyceryl transferase | WD0768 | coding | Full | 792 |
| DNA ligase, NAD-dependent | WD0776 | non-coding | Full | 1864 |
| lipoyl synthase | WD0392 | non-coding | Full | 864 |
| lipoprotein signal peptidase | WD0760 | coding | Full | 477 |
| lysyl-tRNA synthetase | WD0860 | non-coding | Full | 1534 |
| M16 family peptidase putative | WD0737 | coding | Full | 1272 |
| M16 family peptidase putative | WD0737 | coding | Full | 398 |
| M48 family peptidase | WD0652 | non-coding | Full | 1259 |
| maf protein | WD0333 | coding | Full | 597 |
| membrane-associated zinc metalloprotease, putative | WD1086 | coding | Full | 1109 |
| methyltransferase, putative | WD1091 | coding | Full | 666 |
| tRNA delta(2)-isopentenylpyrophosphate transferase | WD0822 | coding | Full | 873 |
| phospho-N-acetylmuramoyl-pentapeptide- transferase | WD1102 | non-coding | Full | 1030 |
| UDP-N-acetylenolpyruvoylglucosamine reductase | WD0541 | non-coding | Full | 839 |
| UDP-N-acetylmuramoylalanine--D-glutamate ligase | WD0849 | non-coding | Full | 1529 |
| NifR3 family protein | WD0025 | non-coding | Full | 584 |
| nuclease-related protein | WRi_012180 | non-coding | Full | 514 |
| NADH dehydrogenase subunit E | WD0734 | coding | Full | 501 |
| transcription antitermination protein NusG, putative | WD0019 | coding | Full | 832 |
| peptidase, M22 family protein | WD0699 | coding | Full | 1014 |
| peptidyl-prolyl cis-trans isomerse D, putative | WD0797 | non-coding | Full | 1809 |
| ubiquinol-cytochrome c reductase, cytochrome b | WRi_011070 | coding | Full | 1230 |
| ubiquinol-cytochrome c reductase, cytochrome c1 | WRi_011060 | coding | Full | 759 |
| phosphate ABC transporter, permease protein, putative | WD0202 | non-coding | Full | 740 |
| phosphatidylglycerophosphatase A, putative | WD0730 | non-coding | Full | 482 |
| phosphoribosylglycinamide formyltransferase, putative | WD0763 | coding | Full | 561 |
| pyruvate phosphate dikinase | WD0690 | non-coding | Full | 2807 |
| prophage LambdaW1, baseplate assembly protein J,putative | WD0283 | coding | Full | 533 |
| prophage LambdaW5, site-specific recombinase resolvase family protein | WD0634 | coding | Full | 367 |
| prophage LambdaW5, baseplate assembly protein W,putative | WD0640 | coding | Full | 336 |
| prophage LambdaW5, baseplate assembly protein V | WD0642 | non-coding | Full | 716 |
| ribose-phosphate pyrophosphokinase | WRi_000300 | coding | Full | 932 |
| adenylosuccinate lyase | WD0786 | coding | Full | 1290 |
| phosphoribosylamine--glycine ligase | WD0029 | coding | Full | 1272 |
| phosphoribosylaminoimidazolecarboxamide formyltransferase/IMP cyclohydrolase | WD0867 | coding | Full | 1512 |
| phosphoribosylaminoimidazole carboxylase, ATPasesubunit | WD1142 | non-coding | Full | 1065 |
| bifunctional proline dehydrogenase/pyrroline-5-carboxylate dehydrogenase | WD0103 | non-coding | Full | 1540 |
| putative monovalent cation/H+ antiporter subunitD | WD1081 | non-coding | Full | 1556 |
| putative monovalent cation/H+ antiporter subunitE | WD0765 | coding | Full | 375 |
| CTP synthetase | WD0468 | non-coding | Full | 1603 |
| DNA repair protein RadC | WD0357 | coding | Full | 678 |
| ATP-dependent DNA helicase RecG | WRi_007900 | non-coding | Partial | 1494 |
| ATP-dependent DNA helicase RecG | WRi_007900 | non-coding | Partial | 533 |
| single-stranded-DNA-specific exonuclease RecJ | WD0312 | non-coding | Full | 1743 |
| transcription termination factor Rho | WD0795 | coding | Full | 1404 |
| riboflavin biosynthesis protein RibD | WD0710 | non-coding | Full | 1062 |
| riboflavin synthase subunit alpha | WD0130 | non-coding | Full | 420 |
| riboflavin biosynthesis protein RibF | WD0759 | coding | Full | 877 |
| ribonucleotide-diphosphate reductase beta subunit | WRi_002040 | coding | Full | 990 |
| ribosomal binding factor A | WD1317 | non-coding | Full | 399 |
| ribosomal protein L20 | WD0865 | non-coding | Full | 569 |
| ribonuclease III | WD1240 | coding | Full | 705 |
| ribonuclease HII | WD1103 | coding | Full | 597 |
| ribulose-phosphate 3-epimerase | WD0712 | non-coding | Full | 678 |
| 50S ribosomal protein L1 | WD0021 | coding | Full | 654 |
| 50S ribosomal protein L2 | WD0678 | coding | Full | 825 |
| 50S ribosomal protein L3 | WD0681 | non-coding | Full | 724 |
| 50S ribosomal protein L4 | WD0680 | non-coding | Full | 614 |
| 50S ribosomal protein L9 | WD0783 | coding | Full | 548 |
| 50S ribosomal protein L10 | WD0022 | non-coding | Full | 511 |
| 50S ribosomal protein L11 | WD0020 | coding | Full | 268 |
| 50S ribosomal protein L7/L12 | WD0023 | non-coding | Full | 404 |
| 50S ribosomal protein L17 | WD0657 | non-coding | Full | 429 |
| 50S ribosomal protein L20 | WD0865 | non-coding | Full | 310 |
| 50S ribosomal protein L22 | WD0676 | non-coding | Full | 355 |
| 50S ribosomal protein L23 | WD0679 | coding | Full | 288 |
| 50S ribosomal protein L28 | WD0391 | coding | Full | 189 |
| ribosomal protein L34 | WRi_001900 | coding | Full | 135 |
| ribosomal protein L35 | WD0864 | coding | Full | 207 |
| ribosomal protein L36 | WRi_004460 | coding | Full | 129 |
| DNA-directed RNA polymerase, beta/beta' subunits | WD0024 | non-coding | Full | 8484 |
| heat shock sigma factor RpoH | WRi_011000 | coding | Full | 813 |
| 30S ribosomal protein S1 | WD1090 | non-coding | Full | 1645 |
| 30S ribosomal protein S6 | WD0781 | non-coding | Full | 597 |
| 30S ribosomal protein S10 | WD0682 | coding | Full | 321 |
| 30S ribosomal protein S11 | WD0659 | non-coding | Full | 448 |
| 30S ribosomal protein S13 | WD0660 | non-coding | Full | 133 |
| 30S ribosomal protein S16 | WD0798 | coding | Full | 321 |
| 30S ribosomal protein S19 | WD0677 | non-coding | Full | 286 |
| ribosomal protein S20 | WRi_009880 | non-coding | Full | 257 |
| crossover junction endodeoxyribonuclease RuvC | WD0142 | non-coding | Full | 289 |
| SCO1/SenC family protein | WD0109 | non-coding | Full | 546 |
| preprotein translocase subunit SecB | WD0106 | non-coding | Full | 378 |
| preprotein translocase subunit SecB | WD0106 | non-coding | Full | 347 |
| preprotein translocase subunit SecG | WD0467 | coding | Full | 369 |
| seryl-tRNA synthetase | WD0028 | non-coding | Full | 1299 |
| site-specific recombinase, phage integrase family | WRi_009900 | non-coding | Full | 973 |
| small protein A, tmRNA-binding | WRi_002050 | coding | Full | 339 |
| SsrA-binding protein | WD0767 | non-coding | Full | 466 |
| superoxide dismutase, Fe | WD0738 | non-coding | Full | 376 |
| sodium/alanine symporter family protein, putative | WD0330 | non-coding | Full | 1124 |
| single-strand binding protein | WD0774 | non-coding | Full | 483 |
| Sua5/YciO/YrdC/YwlC family protein | WD0820 | non-coding | Full | 589 |
| sugE protein | WD0100 | non-coding | Full | 231 |
| surface antigen | WD1085 | non-coding | Full | 1337 |
| surface antigen | WD1085 | coding | Full | 1026 |
| Surface antigen | WD1063 | Non-coding | Partial | 64 |
| TenA family transcription regulator | WD0140 | coding | Full | 684 |
| TenA family transcription regulator | WD0139 | coding | Full | 588 |
| queuine tRNA-ribosyltransferase | WD0735 | non-coding | Full | 1048 |
| transketolase | WD0387 | non-coding | Full | 2038 |
| tldD protein | WD0998 | coding | Full | 1413 |
| outer membrane protein TolC, putative | WD0068 | non-coding | Full | 501 |
| triosephosphate isomerase | WD0091 | non-coding | Full | 626 |
| transposase, degenerate | WD0907 | non-coding | Full | 379 |
| transposase | WRi_008070 | coding | Full | 303 |
| transposase, degenerate | WRi_p08080 | non-coding | Full | 813 |
| transposase, degenerate | WRi_p04500 | non-coding | Full | 745 |
| transposase, degenerate; ISWpi3 | WRi_p03810 | non-coding | Partial | 1051 |
| transposase, degenerate | WD0907 | non-coding | Full | 268 |
| tryptophanyl-tRNA synthetase | WD0801 | coding | Full | 958 |
| tRNA pseudouridine synthase A | WRi_011040 | non-coding | Full | 731 |
| elongation factor Tu | WD0017 | coding | Full | 584 |
| elongation factor Tu | WD0017 | coding | Full | 543 |
| type I secretion system ATPase | WD0770 | coding | Full | 1701 |
| type IV secretion system protein VirB11 | WRi_000080 | non-coding | Full | 992 |
| ubiquinone/menaquinone biosynthesis methlytransferase UbiE | WD0393 | non-coding | Full | 715 |
| phenylacrylic acid decarboxylase, 3-octaprenyl-4-hydroxybenzoate carboxy-lyase | WD0556 | coding | Full | 576 |
| excinuclease ABC subunit B | WD0839 | non-coding | Full | 1916 |
| type IV secretion system protein VirB10 | WD_0006 | coding | Full | 441 |
| type IV secretion system ATPase VirB4 | WD0858 | non-coding | Full | 2409 |
| type IV secretion system protein VirB6 | WD0857 | coding | Full | 1033 |
| type IV secretion system protein VirB6 | WD0857 | coding | Full | 309 |
| type IV secretion system protein VirB8 | WD0004 | coding | Full | 456 |
| type IV secretion system protein VirB9 | WD0005 | non-coding | Full | 792 |
| type IV secretion system protein VirB10 | WD_0006 | non-coding | Full | 722 |
| 23S ribosomal RNA | WRi_r01850 | rRNA | rRNA | 1601 |
| ribosomal RNA large subunit methyltransferase J,putative | WD0070 | rRNA | rRNA | 662 |
| tRNA-Ala | WRi_t09220 | tRNA | tRNA | 73 |
| tRNA-Arg | WRi_t00350 | tRNA | tRNA | 78 |
| tRNA-Arg | WRi_t06170 | tRNA | tRNA | 74 |
| tRNA-Gly | WRi_t05220 | tRNA | tRNA | 71 |
| tRNA-Leu | WRi_t04010 | tRNA | tRNA | 87 |
| tRNA-Leu | WRi_t09470 | tRNA | tRNA | 85 |
| tRNA-Lys | WRi_t09230 | tRNA | tRNA | 73 |
| tRNA-Met | WRi_t06270 | tRNA | tRNA | 64 |
| tRNA-Ser | WRi_t07290 | tRNA | tRNA | 87 |
| tRNA-Thr | WRi_t01600 | tRNA | tRNA | 71 |
| tRNA-Trp | WRi_t00160 | tRNA | tRNA | 73 |
| tRNA-Tyr | WRi_t05230 | tRNA | tRNA | 83 |
