# Supplementary material for: Presence of Extensive Wolbachia Symbiont Insertions Discovered in the Genome of Its Host Glossina morsitans morsitans
Source: PLoS Negl Trop Dis. 2014 Apr 24;8(4):e2728. doi: 10.1371/journal.pntd.0002728 (PMC3998919; doi:10.1371/journal.pntd.0002728)
Supplement: Table S8 — Description of the third set of Wolbachia inserted regions into the G. m. morsitans chromosomes. (DOCX) [file pntd.0002728.s012.docx]

**Table S8.** Description of the third set of *Wolbachia* inserted regions (Insertion C) into the *G. m. morsitans* chromosomes.

| **Product** | **Homolog** | **Type** | **Insertion** | **Length** |
| --- | --- | --- | --- | --- |
| 3,4-dihydroxy-2-butanone 4-phosphate synthase, | WD_0653 | Non-coding | Partial | 245 |
| 30S ribosomal protein S4_rpsD | WD_0388 | Non-coding | Partial | 153 |
| NifU domain-containing protein | WD_1075 | Non-coding | Partial | 191 |
| ATP-dependent protease La_2247 | WD0317 | Non-coding | Partial | 500 |
| Hypothetical protein | WD0728 | Non-coding | Partial | 500 |
| preprotein translocase subunit SecB | WD_0103 | Non-coding | Partial | 347 |
